# Quantitative μ-Opioid Receptor PET with [^18^F]FCFN in Nonhuman Primates: Naloxone Blockade and Within-Scan Displacement

**DOI:** 10.3390/pharmaceutics18091141

**Published:** 2026-09-10

**Authors:** Evan Gallagher, Joseph W. Downey, Chi-Hyeon Yoo, Hongping Deng, Jessie Fanglu Fu, Qikai Qin, Irena Bass, Hsiao-Ying Wey, Jacob M. Hooker, Christin Y. Sander, So Jeong Lee

**Affiliations:** Athinoula A. Martinos Center for Biomedical Imaging, Department of Radiology, Massachusetts General Hospital and Harvard Medical School, Charlestown, MA 02129, USA

**Keywords:** μ-opioid receptor, PET imaging, radiotracer evaluation, [^18^F]fluorocarfentanil ([^18^F]FCFN), nonhuman primate

## Abstract

**Background/Objectives**: Quantitative PET imaging of μ-opioid receptor (MOR) availability and pharmacologic engagement can advance in vivo studies of opioid receptor biology. We previously evaluated a series of ^18^F-labeled carfentanil analogs and prioritized the regioisomer [^18^F]fluorocarfentanil ([^18^F]FCFN) based on favorable brain kinetics, regional distribution, and naloxone-sensitive binding. Here, we provide proof-of-concept evaluation of [^18^F]FCFN in rhesus macaques to assess its potential for quantitative, pharmacologically responsive MOR PET. **Methods**: [^18^F]FCFN was produced by automated radiosynthesis. Dynamic [^18^F]FCFN PET scans were performed in two rhesus macaques under baseline conditions, after low- (0.005 mg/kg) and high-dose (0.2 mg/kg) naloxone pretreatment, and during within-scan intravenous or intranasal naloxone challenge. For arterial input scans, two-tissue compartment modeling with metabolite-corrected arterial input functions was used to estimate total distribution volume (*V*_T_). Nondisplaceable binding potential (BP_ND_) was estimated using multilinear reference tissue model 2 with the cerebellum as the candidate reference region. **Results**: Automated radiosynthesis produced [^18^F]FCFN in high radiochemical yield and molar activity, enabling low-mass PET injections (0.09–0.32 μg). [^18^F]FCFN showed rapid brain uptake and MOR-consistent regional distribution, with lowest uptake in the cerebellum. Regional BP_ND_ correlated positively with *V*_T_ at baseline (R^2^ = 0.983, *p* < 0.0001) and after high-dose blockade (R^2^ = 0.522, *p* < 0.004). Naloxone pretreatment reduced regional BP_ND_ in a dose-dependent manner. Within-scan intravenous and intranasal naloxone challenges also produced marked reductions in BP_ND_. **Conclusions**: [^18^F]FCFN enables quantitative, pharmacologically sensitive MOR PET imaging in nonhuman primates at low injected masses. Concordant plasma-input and reference-tissue measures, dose-dependent blockade, and proof-of-concept within-scan displacement support its use for measuring MOR availability and dynamic antagonist engagement across administration routes.

## 1. Introduction

The μ-opioid receptor (MOR) is the molecular target through which endogenous opioid peptides, prescription analgesics, and illicit opioids exert their effects on nociception, reward, affect, and respiratory drive [1,2,3,4]. MOR availability is known to be altered by pain, substance use, affective state, withdrawal, and pharmacologic challenge [4,5,6,7,8,9,10]. Because behavioral measures and peripheral biomarkers cannot localize or quantify these changes in the living brain, PET imaging of the MOR has become a critical in vivo readout for opioid neurobiology.

**Figure 1:**
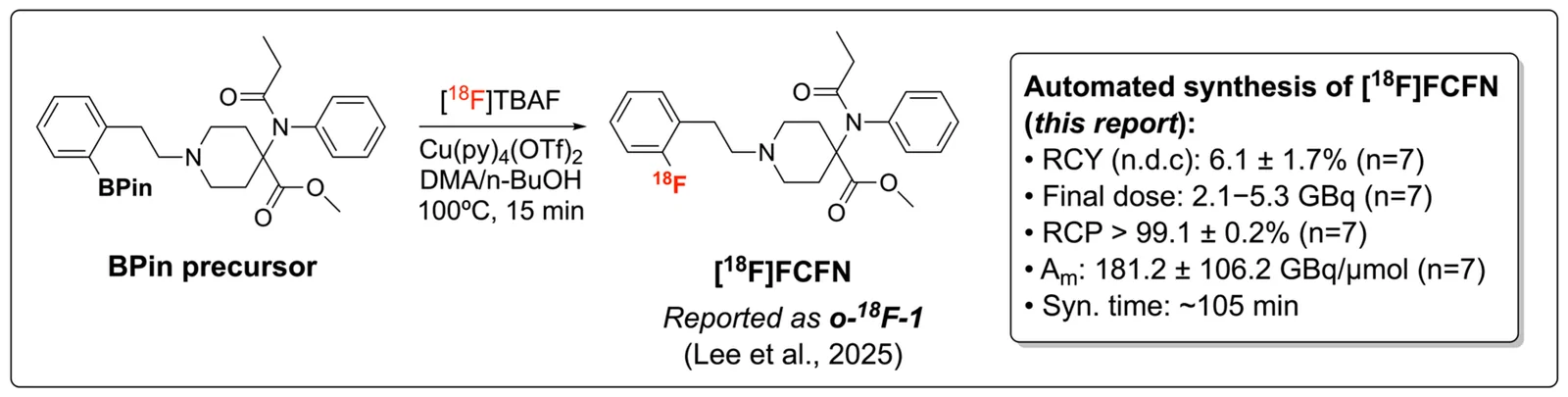
Automated radiosynthesis of [^18^F]FCFN. The radiosynthetic scheme for [^18^F]FCFN, previously reported as *o*-^18^F-1, was described in our prior work [17]; the automated synthesis results shown here are from the present study. RCY = radiochemical yield; n.d.c = non-decay corrected; RCP = radiochemical purity; A_m_ = molar activity.

[^11^C]Carfentanil ([^11^C]CFN) remains the benchmark MOR-selective PET agonist tracer and has supported a generation of human studies on receptor availability, endogenous opioid release, pain modulation, craving, and antagonist occupancy [5,6,7,8,9,10,11,12]. Its broader use, however, is limited by two practical constraints: the 20 min half-life of carbon-11, which restricts use to sites with on-site cyclotron and radiochemistry infrastructure, and the high MOR agonist potency of carfentanil, which narrows the allowable injected-mass range even under tracer-dose conditions [13]. These constraints can limit experimental flexibility, particularly for extended or repeated imaging. Alternative opioid radiotracers such as [^11^C]diprenorphine and [^18^F]FE-DPN broaden experimental options but lack MOR selectivity and, as antagonists, exhibit different binding characteristics from agonist-based tracers [14,15,16]. To address these limitations, we recently reported a series of ^18^F-labeled carfentanil analogs and identified [^18^F]fluorocarfentanil ([^18^F]FCFN; Figure 1) as a leading MOR PET tracer candidate in rats, with favorable brain uptake, a regional distribution consistent with known MOR expression, and naloxone-sensitive binding [17]. FCFN showed approximately 35-fold lower MOR potency (K_i_ = 0.7 nM) than carfentanil, while selective KOR and DOR blockade studies further supported its MOR selectivity [17].

A key next translational step is to determine whether [^18^F]FCFN can support quantitative and pharmacologically responsive MOR imaging in the primate brain. This capability is particularly relevant to naloxone pharmacology and opioid overdose reversal. Intranasal naloxone is widely used for emergency opioid reversal, yet its central pharmacodynamics remain incompletely characterized. Important questions include the timing and regional extent of brain MOR engagement and how these depend on antagonist dose and route; these questions have been examined directly in only one published human PET study [11]. Although bolus-plus-infusion paradigms have been implemented with [^11^C]CFN, prolonged within-scan pharmacologic challenge studies remain constrained by carbon-11 decay and the stringent injected-mass requirements of carfentanil. The longer half-life of fluorine-18, together with the lower MOR potency of FCFN [17], may therefore provide greater flexibility for prolonged bolus-plus-infusion imaging and dynamic assessment of antagonist engagement.

Here, we extend the evaluation of [^18^F]FCFN from rodents to nonhuman primates (NHPs). This study was designed as an initial NHP feasibility evaluation rather than a statistically powered comparison across experimental conditions, with the goals of establishing automated production of [^18^F]FCFN, characterizing its brain kinetics and quantitative imaging properties in rhesus macaques, and assessing pharmacologic sensitivity to naloxone. We evaluated blockade across low- and high-dose intravenous naloxone pretreatment conditions and performed proof-of-concept bolus-plus-infusion experiments to assess within-scan displacement after intravenous and intranasal naloxone challenge. The intranasal route was specifically selected because it represents the clinically deployed route of naloxone administration for emergency opioid reversal and provides an absorption-dependent challenge distinct from direct intravenous delivery, allowing us to test whether [^18^F]FCFN could capture dynamic MOR engagement after a clinically relevant nonintravenous route.

## 2. Materials and Methods

### 2.1. Chemicals and Reference Standards

The nonradioactive reference compound FCFN and the corresponding aryl boronic ester precursor (BPin precursor, Figure 1) were synthesized as previously described [17].

### 2.2. Radiosynthesis of [^18^F]FCFN

Automated production of [^18^F]FCFN was adapted from the previously reported radiosynthesis [17] and implemented on a Trasis AllInOne (AIO 30) (Trasis SA, Ans, Belgium) automated synthesis module using a modified commercial cassette (Figure S1). Detailed cassette configuration, reagent quantities, HPLC conditions, and quality-control procedures are provided in Supplementary Materials Section S1 (Radiochemistry) and Figures S1–S3.

### 2.3. Animals

All procedures were approved by the Institutional Animal Care and Use Committee at Massachusetts General Hospital (IACUC# 2003N000338) and were conducted in accordance with institutional regulations and ARRIVE reporting guidelines [18]. Two adult male rhesus macaques (ages 12–13, 7.9–11.0 kg) were imaged for this study. Anesthesia was induced with ketamine/xylazine (10 mg/kg and 0.5 mg/kg, respectively), and maintained with isoflurane (1–1.5%) vaporized in pure oxygen. Heart rate, respiratory rate, blood pressure, end-tidal CO_2_, and oxygen saturation were monitored throughout the imaging session. The animal assigned to each scan is provided in Supplementary Materials Section S2 and Table S1.

### 2.4. Naloxone Administration

For pretreatment studies, naloxone was administered intravenously at low and high doses (0.005 and 0.2 mg/kg, respectively) 10 min before [^18^F]FCFN injection. For within-scan displacement studies, [^18^F]FCFN was administered using a bolus-plus-infusion protocol, and naloxone was administered during the scan either intravenously (0.2 mg/kg) or intranasally (0.4 mg/kg) at 75 min after tracer injection. Naloxone doses, formulations, and administration volumes are provided in the Supplementary Materials Section S3.

### 2.5. Arterial Input Function, Radiometabolite Analysis, and Plasma Free Fraction

For arterial input studies, arterial blood samples were collected at 26 timepoints throughout two independent PET scans (Table S1), one under baseline conditions and one following high-dose naloxone blockade (0.2 mg/kg). Selected plasma samples were analyzed by radio-HPLC to determine the fraction of unchanged parent radiotracer and the radiometabolite profile (see Supplementary Materials Section S3) [19]. The measured parent fractions were used to generate the metabolite-corrected arterial plasma input function for kinetic modeling. The plasma free fraction (*f*_P_) was measured by ultrafiltration [20] to characterize plasma protein binding of [^18^F]FCFN. Radiotracer was added to plasma, incubated at room temperature, and centrifuged through ultrafiltration devices. *f*_P_ was calculated as the ratio of radioactivity concentration in the ultrafiltrate to that in total plasma. Detailed blood-processing, radio-HPLC, and ultrafiltration procedures, together with the measured *f*_P_ value, are provided in Supplementary Materials Section S3.

### 2.6. PET/MR Study Design and Acquisition

Seven dynamic [^18^F]FCFN scans were performed: five using bolus injection and two using bolus-plus-infusion administration of tracer. For bolus studies, imaging was performed on a BrainPET insert within a 3T magnetic resonance (MR) scanner (Tim Trio, Siemens Healthineers, Erlangen, Germany) [21,22]. T1-weighted MR images were acquired before tracer injection for anatomic registration [23]. Animals received 69.19–76.22 MBq (1.87–2.06 mCi) of [^18^F]FCFN intravenously, followed by dynamic PET acquisition for 120 min. For bolus-plus-infusion studies, imaging was performed on a Siemens Biograph mMR PET/MR scanner (Siemens Healthineers, Erlangen, Germany). As with the bolus studies, a T1-weighted anatomical MRI was acquired prior to tracer injection. For the within-scan displacement studies, 38.11–52.54 MBq (1.03–1.42 mCi) of [^18^F]FCFN containing 0.09–0.32 μg cold mass was administered as a bolus and 37.37–51.80 MBq (1.01–1.40 mCi) containing 0.44–0.57 μg cold mass was administered as continuous infusion over a 140 min scan (bolus-to-infusion activity ratio = 1:1, K_bol_ = 140 min). Consistent with previous bolus-plus-infusion receptor PET studies [24,25], stabilization of regional activity before pharmacological challenge using naloxone was assessed from regional time–activity curves 60–75 min after tracer administration. The pre-challenge plateau was operationally defined as an absolute change of ≤5% in fitted regional standardized uptake values (SUV) over this 15 min interval. Following naloxone challenge at 75 min, the time–activity curves (TACs) were analyzed dynamically to characterize displacement, including time to 50% displacement and magnitude of displacement at the end of the 140 min acquisition.

### 2.7. Image Processing and Region-of-Interest Analysis

For the bolus-only studies, PET images were reconstructed using a standard 3D Poisson ordered subset expectation maximization (OSEM) algorithm using prompt and variance-reduced random coincidence events. Normalization, scatter, and attenuation sinograms were also included in the reconstruction [26], and the reconstructed images consisted of 1.25 × 1.25 × 1.25 mm voxels in a 128 × 128 × 76 matrix. The dynamic frame times were as follows: 4 × 15 s, 4 × 30 s, 4 × 60 s, 8 × 120 s, 19 × 300 s. For the bolus-plus-infusion studies, PET images were reconstructed using manufacturer-supplied software. Three iterations and 21 subsets were used for these reconstructions, and normalization and scatter correction were performed according to manufacturer recommendations. By default, the Biograph mMR system uses an MR-based attenuation correction approach based on the DIXON MR sequence [27,28]. However, this approach is optimized for human subjects, and DIXON-based attenuation maps did not align well with NHP neck and head anatomy. To address this issue, we registered preexisting CT images from each animal to that animal’s DIXON MR image via the imregtform function in Matlab 2025b (The MathWorks, Inc., Natick, MA, USA). We then used k-means clustering to generate animal-specific CT-based attenuation maps for each of the two bolus-plus-infusion studies. The resulting reconstructed images consisted of 2.086 × 2.086 × 2.031 mm voxels in a 344 × 344 × 127 matrix. The dynamic frame times were identical to those used in the bolus-only studies, except that twenty-four 300 s frames were used instead of nineteen. Reconstructed dynamic PET images were motion corrected using the mc-afni2 script supplied with Freesurfer 8.1.0 [29,30], and NiftyReg 1.3.9 [31,32] was then used to perform affine registration of the PET image to the corresponding T1 MRI. NiftyReg was then used again to register the T1 MRI to the WADRC 112RM-SL rhesus macaque brain template [33], and the resulting transformation was applied to the motion-corrected PET image to generate the final dynamic PET image used for analysis. Fourteen regions-of-interest (ROIs) from the NeuroMaps atlas [34] were overlaid onto this final PET image and used to generate the TACs for kinetic modeling. For regional TACs, SUV was calculated as tissue radioactivity concentration (Bq/mL) divided by injected activity per unit body weight (Bq/g) and reported in g/mL consistent with the body-weight-normalized SUV convention.

### 2.8. Kinetic Modeling and Outcome Measures

For arterial input function-based kinetic modeling, we utilized one-tissue (1TC) and two-tissue compartment (2TC) models, using the Akaike information criterion (AIC) to assess goodness of model fit. For both models, regional *V*_T_ was used as the primary outcome measure [35]. For comparison, *V*_T_ was also calculated using multilinear analysis-1 (MA1), and agreement between MA1- and 2TC-derived *V*_T_ values was assessed. For studies that did not involve arterial blood sampling, the cerebellum and visual cortex were evaluated as candidate reference-tissue regions based on their previous use in [^11^C]CFN studies [36,37]. Candidate regions were compared using regional SUVs, washout kinetics, and the magnitude of naloxone-associated changes in uptake. The region showing the more favorable reference-tissue characteristics was then used as the reference region for multilinear reference tissue model 2 (MRTM2) [38]. BP_ND_ was estimated using MRTM2 and compared with plasma-input *V*_T_ under baseline and blocked conditions. Model fitting was performed using PMOD version 4.006 (for 1TC, 2TC, and MA1 models) or Fastmap version 3.0 (for the MRTM2).

### 2.9. Statistical Analysis

Regional outcome measures are reported as mean or mean ± range where appropriate. Associations between modeling outcomes were assessed using linear regression. Percent blockade and displacement were calculated relative to baseline or pre-challenge values as specified. Statistical significance was defined as *p* < 0.05, and all statistical analyses were performed in GraphPad Prism version 11.0.1.

## 3. Results

### 3.1. Automated Radiosynthesis of [^18^F]FCFN

[^18^F]FCFN was produced using an automated synthesis module with integrated radiolabeling, semipreparative HPLC purification, solid-phase extraction, and sterile reformulation. The total synthesis time was 105 min from end-of-bombardment (EOB) to end-of-synthesis (EOS). Across seven automated productions, [^18^F]FCFN was obtained in 2.1–5.3 GBq (56.3–143.8 mCi) at EOS, with a non-decay-corrected radiochemical yield of 6.1 ± 1.7%, molar activity of 181.2 ± 106.2 GBq/μmol (4.9 ± 2.9 Ci/μmol), and radiochemical purity of 99.1 ± 0.2%. All formulated doses met release criteria for radiochemical purity, pH, residual copper, and chemical purity before injection. Representative semipreparative and analytical HPLC chromatograms are shown in Figure S2.

### 3.2. Baseline Brain Distribution and Kinetics of [^18^F]FCFN

**Figure 2:**
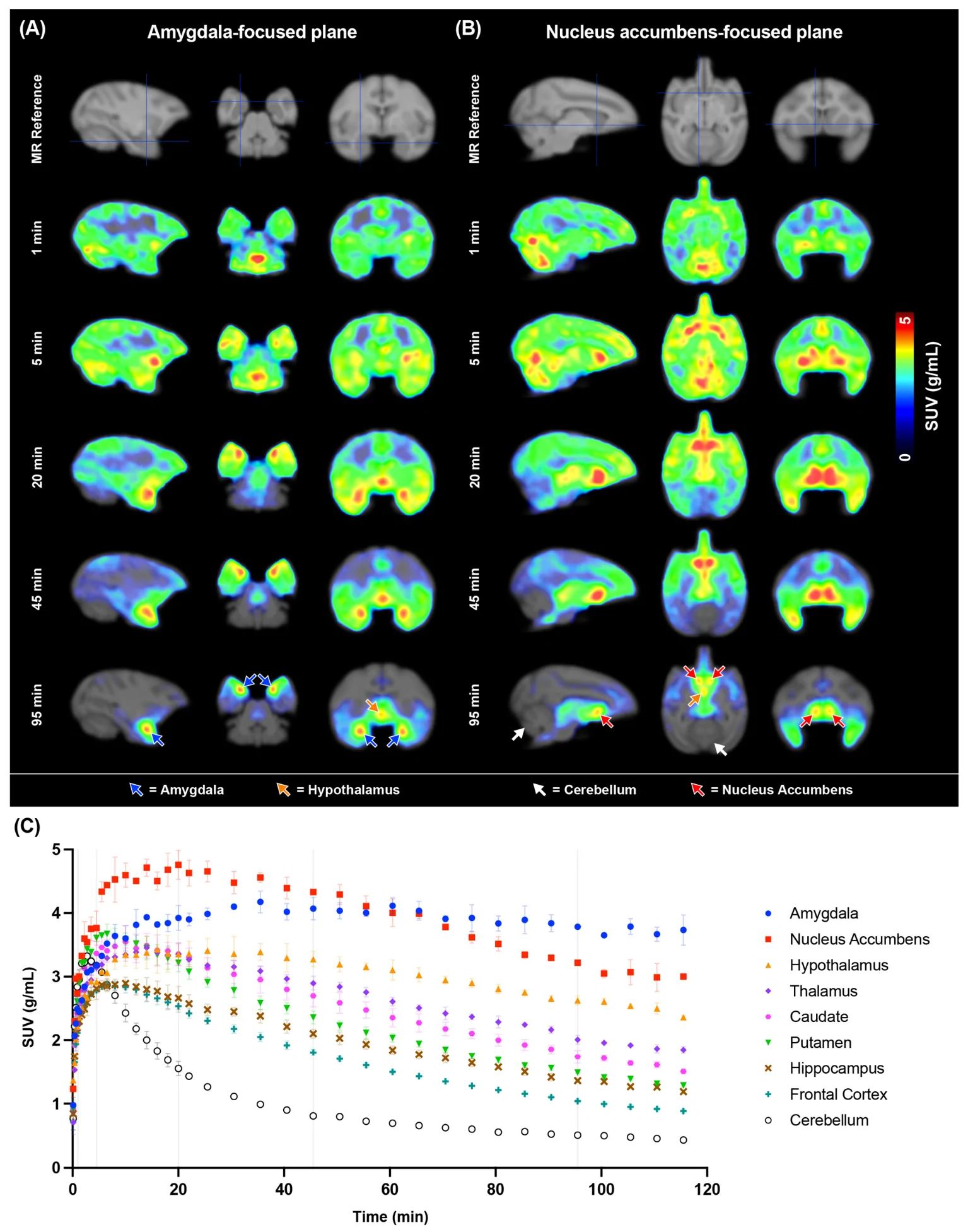
Baseline [^18^F]FCFN PET images and regional time–activity curves. (**A**,**B**) Average [^18^F]FCFN PET images from two baseline scans in the same rhesus macaque at selected timepoints. The anatomical locations and boundaries of the nine key brain regions evaluated in this study are illustrated on a rhesus macaque MRI atlas in Figure S4. Images are displayed in planes centered on the amygdala (**A**) and nucleus accumbens (**B**), and are overlaid onto a population-average T1-weighted rhesus macaque MRI template [33]. PET images are shown in standardized uptake value (SUV) units, and crosshairs on the MR template indicate the corresponding PET image planes. In the bottom row of images, arrows indicate the amygdala (blue), hypothalamus (orange), and nucleus accumbens (red). (**C**) Regional time–activity curves (TACs). Here, colored icons show average regional SUVs from two baseline scans, while colored bars show the range. Additional gray vertical lines indicate the timepoints for the PET images shown in (**A**,**B**).

Baseline dynamic PET scans were acquired to characterize the regional distribution and kinetics of [^18^F]FCFN in the rhesus macaque brain (Figure 2). Representative PET images are shown in Figure 2A,B, and average TACs from nine ROIs are shown in Figure 2C. For each ROI, quantitative SUV metrics including average SUV from 60 to 120 min (SUV_60–120_), peak SUV, time to peak SUV, SUV at 120 min as a percentage of peak, and time to 50% washout measures, are summarized in Table S2.

[^18^F]FCFN showed heterogeneous brain uptake across regions. The nucleus accumbens (NAC) showed the highest peak SUV (4.76), whereas the amygdala showed the highest SUV_60–120_ (3.85). These regions also differed in kinetic profile: the NAC peaked earlier (20 min) and showed 37% washout by the end of the scan, whereas the amygdala peaked later (35.5 min) and showed the slowest washout among the regions examined. Moderate uptake and retention were observed in hypothalamus, thalamus, caudate, putamen, and anterior cingulate cortex (ACC). The cerebellum showed the lowest uptake and fastest washout among the evaluated regions, consistent with the known scarcity of MORs in this region [17,36,37,39].

A single baseline scan with the related regioisomer *p*-^18^F-2 (Figure S3) was also acquired because this compound had shown promising in vivo characteristics in our previous rodent study [17]. This supplementary evaluation showed a broadly similar regional uptake pattern, including low cerebellar uptake, but generally slower uptake and washout kinetics than [^18^F]FCFN (Figure S5). Low cerebellar uptake was therefore observed with both related regioisomers, although cerebellar clearance was slower with *p*-^18^F-2.

### 3.3. Plasma Input Function and Two-Tissue Compartment Modeling

**Figure 3:**
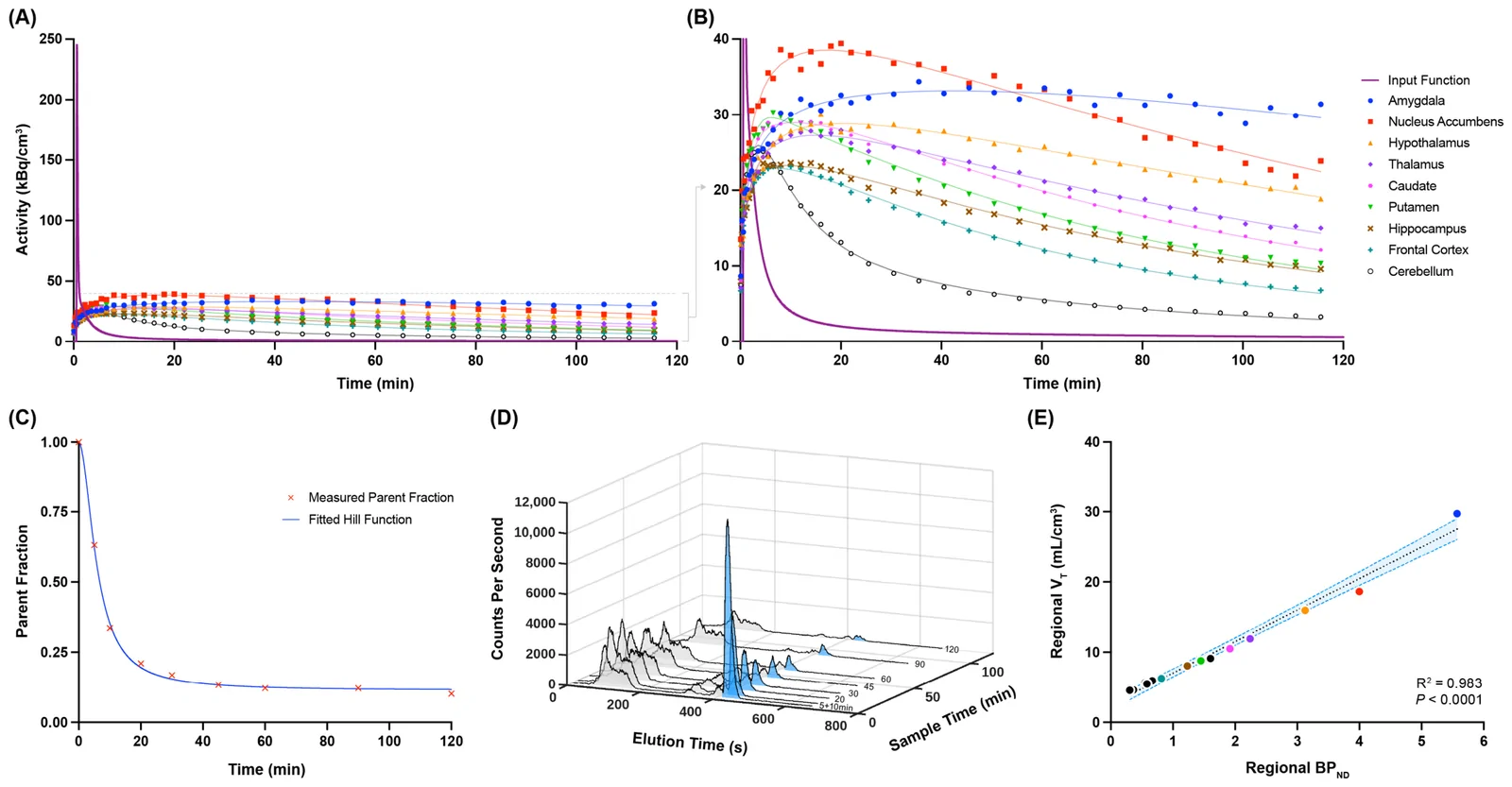
Two-tissue compartment modeling of [^18^F]FCFN at baseline. (**A**,**B**) Regional TACs, metabolite-corrected arterial plasma input function, and two-tissue compartment (2TC) model fits shown at full scale (**A**) and expanded scale (**B**). (**C**) Parent fraction measured by radio-HPLC (red icons) and fitted with a Hill function (blue curve). (**D**) Radio-HPLC chromatograms from post-injection arterial plasma samples showing parent [^18^F]FCFN (blue) and radiometabolites over time. (**E**) Associations between regional *V*_T_ and BP_ND_ under baseline conditions. The shaded band indicates the 95% confidence interval of the linear fit. Here, regional BP_ND_ values were estimated using the multilinear reference tissue model 2 (MRTM2) with the cerebellum as the reference region.

Arterial input studies were performed under baseline conditions and after naloxone pretreatment (0.2 mg/kg) to more robustly quantify [^18^F]FCFN kinetics. Parent-fraction and radiometabolite analyses were performed by radio-HPLC, and metabolite-corrected plasma input functions were generated for kinetic modeling. Model comparison using AIC supported selection of the 2TC model over the 1TC model for both baseline and naloxone-blocked conditions, as the 2TC model consistently showed lower AICs (Table S3). Baseline regional TACs, 2TC model fits, parent-fraction data, and arterial input functions are shown in Figure 3, with corresponding post-naloxone results shown in Figure S6. Regional rate constants and *V*_T_ values are summarized in Table S4.

Under baseline conditions, the two-tissue compartment model provided good fits to regional TACs across evaluated regions (Figure 3A,B). Consistent with the SUV results above, MOR-enriched regions including the amygdala, NAC, and hypothalamus [17,36,37,39], showed the highest average *V*_T_ values (29.7 mL•cm^−3^, 18.6 mL•cm^−3^, and 15.9 mL•cm^−3^, respectively), and the NAC showed the highest peak uptake. Regional *K*_1_ values indicated measurable brain delivery across all evaluated regions, with the NAC showing the highest baseline *K*_1_ value (0.41 mL•cm^−3^•min^−1^) among the regions examined. The amygdala showed slower apparent delivery than the NAC (*K*_1_ = 0.26 mL•cm^−3^•min^−1^ vs. 0.41 mL•cm^−3^•min^−1^), but prolonged late retention on TACs, with the lowest apparent *k*_4_ value (0.019 min^−1^) among all regions at baseline. Parent fraction analysis showed fast metabolism of [^18^F]FCFN during the scan with a 50% reduction in parent fraction by 6.8 min after injection (Figure 3C), whereas the radiometabolite profile remained relatively stable over the imaging period (Figure 3D). The radio-HPLC showed predominantly earlier-eluting, more polar radiometabolites within the first 3 min, together with a small unidentified radioactive peak eluting close to the parent [^18^F]FCFN peak at approximately 7–8 min (Figure 3D). The identity of this minor component was not determined in the present study. Regional brain TACs showed consistent washout without evidence of progressive late accumulation over the acquisition period (Figure 3B). After naloxone pretreatment, fitted 2TC parameters showed reduced tissue retention across regions (Table S4). Some post-naloxone rate constants (*k*_3_ and *k*_4_) approached zero, consistent with markedly reduced specific signal under MOR blockade. Under these conditions, parameter estimates were less stable, with larger associated standard errors.

Regional *V*_T_ estimates obtained from the 2TC model showed excellent agreement with estimates derived from MA1 analysis under baseline conditions (R^2^ = 0.999, *p* < 0.0001; Figure S7A). Under naloxone blocking conditions, regional rank order was preserved, although agreement between 2TC- and MA1-derived *V*_T_ estimates was reduced (R^2^ = 0.626, *p* = 0.0008; Figure S7B), as expected given that MOR blockade reduces specific binding and compresses the regional dynamic range of V_T_ estimates.

### 3.4. Reference Region Selection and Reference-Tissue Quantification

Reference-tissue modeling was next evaluated to support image-based quantification without time- and resource-intensive arterial blood sampling. The cerebellum and visual cortex were assessed as candidate reference regions (Figure S8), as both have previously been used as reference regions in [^11^C]CFN PET studies [17,36,37,40]. Across baseline and naloxone-blocked conditions, the cerebellum showed lower average SUVs than visual cortex, as well as smaller naloxone-associated SUV reductions (8–11% in cerebellum vs. 11–17% in visual cortex), and faster washout (Figure 2C, Figures S5C and S8). These findings indicated a smaller naloxone-sensitive component in the cerebellum than in visual cortex; therefore, the cerebellum was selected as the more appropriate reference region for [^18^F]FCFN reference-tissue modeling in rhesus macaques.

Using the cerebellum as the reference region, regional BP_ND_ values for twelve ROIs were calculated via MRTM2 [38]. Regional BP_ND_ values were positively associated with plasma-input *V*_T_ estimates under both baseline (R^2^ = 0.983, *p* < 0.0001; Figure 3E) and blocked conditions (R^2^ = 0.522, *p* < 0.004; Figure S6E), supporting the use of reference-tissue modeling for subsequent blockade and displacement analyses.

### 3.5. Dose-Dependent Naloxone Blockade

**Figure 4:**
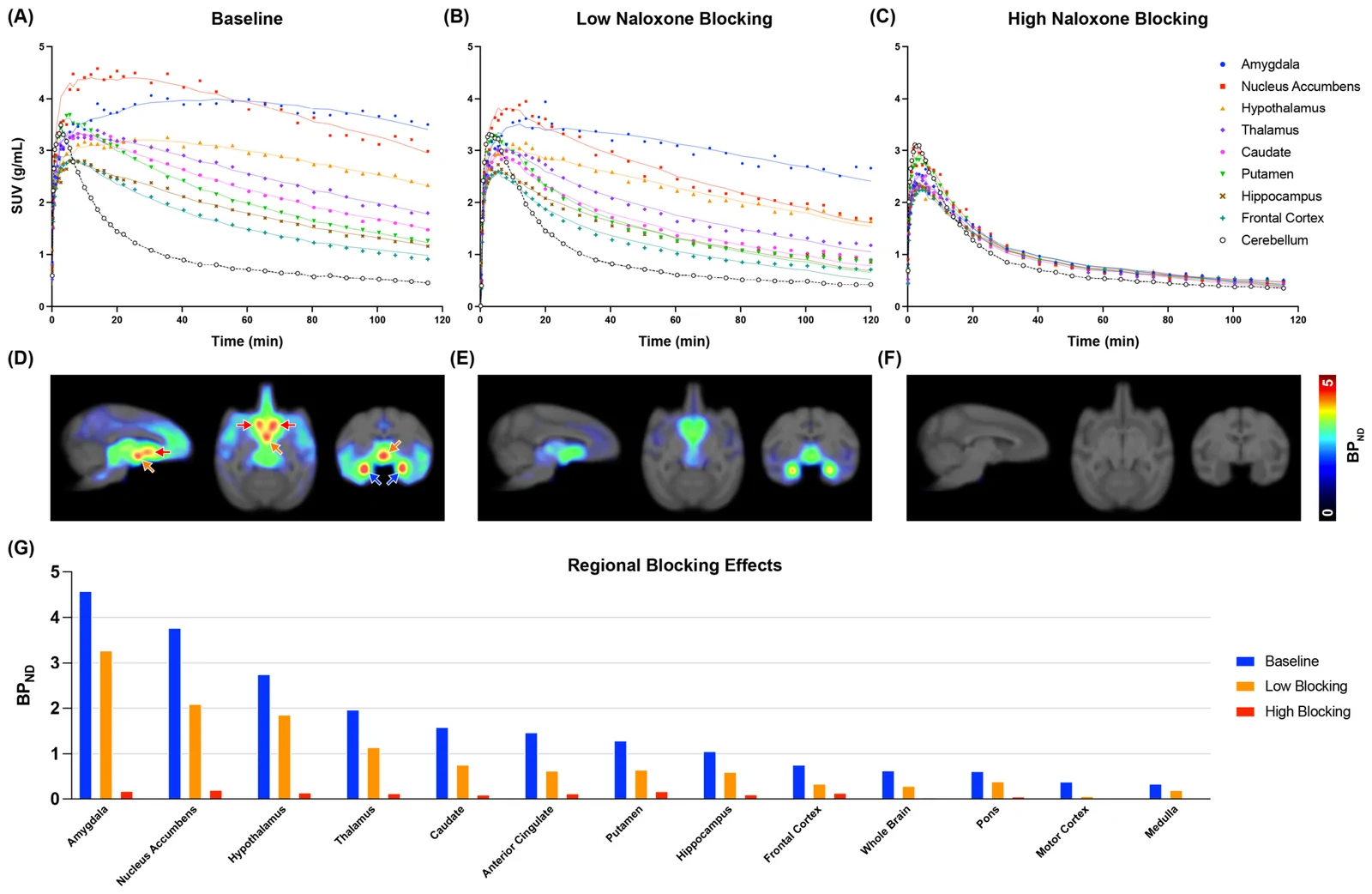
Dose-dependent naloxone blockade of [^18^F]FCFN binding. (**A**–**C**) Regional TACs at baseline (**A**), after low-dose intravenous naloxone pretreatment ((**B**); 0.005 mg/kg), and after high-dose intravenous naloxone pretreatment ((**C**); 0.2 mg/kg). Points indicate SUVs, and solid lines indicate MRTM2 fits using cerebellum as reference region. (**D**–**F**) Corresponding BP_ND_ maps. Arrows indicate amygdala (blue), hypothalamus (orange), and nucleus accumbens (red). (**G**) Regional BP_ND_ values across conditions. Numerical values and percent reductions are shown in Table 1.

Naloxone pretreatment studies were performed to assess the pharmacologic sensitivity of [^18^F]FCFN in vivo. Dynamic PET scans were acquired after low-dose (0.005 mg/kg) and high-dose (0.2 mg/kg) intravenous naloxone pretreatment (Figure 4). Regional BP_ND_ values and percent reductions relative to baseline are summarized in Table 1.

Both naloxone doses reduced regional BP_ND_ values across the brain (Figure 4D vs. Figure 4E,F). Low-dose naloxone reduced regional BP_ND_ by 28–57%, while high-dose naloxone reduced regional BP_ND_ by 58–96% (Figure 4G and Table 1). The marked high-dose blocking effect was observed in both animals. After low-dose naloxone, regions with the highest baseline BP_ND_, including the amygdala, NAC, hypothalamus, and thalamus, continued to show the highest residual BP_ND_ values. After high-dose naloxone, regional BP_ND_ was markedly reduced across evaluated regions. The cerebellum remained the region with the lowest SUV under both baseline (Figure 4A) and naloxone-blocked conditions (Figure 4B,C). While reductions in cerebellar SUV were observed after naloxone pretreatment (8% and 11% in low-dose and high-dose blocking conditions, respectively), this effect was smaller than that observed in any other region.

### 3.6. Within-Scan Displacement by Intravenous and Intranasal Naloxone

**Figure 5:**
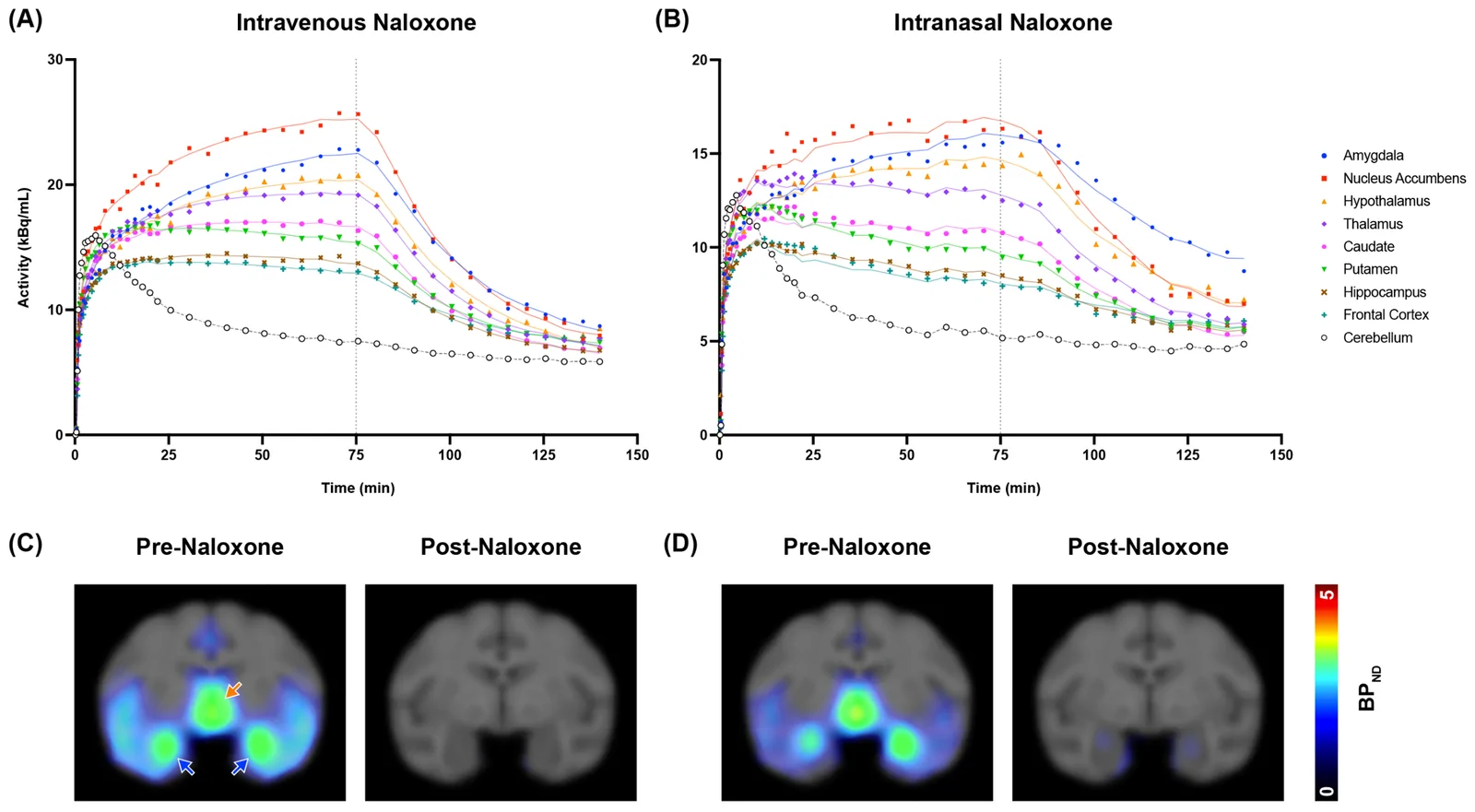
Within-scan displacement of [^18^F]FCFN by intravenous and intranasal naloxone. (**A**,**B**) Regional TACs from bolus-plus-infusion scans before and after intravenous naloxone ((**A**); 0.2 mg/kg) or intranasal naloxone ((**B**); 0.4 mg/kg). [^18^F]FCFN was administered using a bolus-to-infusion ratio of 1:1 (K_bol_ = 140 min), and naloxone was administered 75 min after tracer injection, indicated by the vertical dotted line. (**C**,**D**) BP_ND_ maps before and after intravenous (**C**) or intranasal (**D**) naloxone challenge. Arrows indicate amygdala (blue) and hypothalamus (orange). Regional values and displacement metrics are shown in Table 2.

Within-scan naloxone displacement studies were performed using bolus-plus-infusion administration of [^18^F]FCFN, with naloxone administered at 75 min after radiotracer injection. Naloxone was administered intravenously (IV, 0.2 mg/kg) in one scan and intranasally (IN, 0.4 mg/kg) in the other. Regional TACs and voxel-wise BP_ND_ maps are shown in Figure 5, and displacement measures are summarized in Table 2.

Before naloxone challenge, regional uptake patterns were similar to those observed in bolus baseline scans, with the highest uptake and retention in the NAC, amygdala, hypothalamus, and thalamus. During the 60–75 min pre-challenge interval, approximately 79% of evaluated regional TACs across both scans (Figure 5A,B) met the operational plateau criterion (Section 2.6). Following naloxone administration, most target regions showed marked reductions in BP_ND_ (Figure 5C and Figure 5D, respectively). IV naloxone produced more rapid displacement than IN naloxone, with most regions reaching >50% displacement within 60 min after IV challenge (Table 2). Following IN naloxone, displacement was more gradual, with >50% displacement observed only in selected high-binding regions within the acquisition window (Table 2). Across both routes, high-binding regions generally showed more rapid displacement than moderate- or low-binding regions, with the NAC showing the shortest time to 50% displacement.

The amygdala retained the highest late-scan SUV after naloxone challenge despite lower earlier uptake than the NAC. This NAC–amygdala crossover was observed across dynamic scans (Figure 2, Figure 3, Figure 4 and Figure 5) and was consistent with slower apparent [^18^F]FCFN washout in the amygdala. Naloxone challenge produced minimal changes in cerebellar TAC slope relative to target regions (Figure 5A,B), consistent with a small naloxone-sensitive component in the reference region.

## 4. Discussion

This study extends the characterization of [^18^F]FCFN, an ^18^F-labeled MOR agonist radiotracer previously reported in rats, to NHPs. [^18^F]FCFN showed a regional brain distribution consistent with known MOR enrichment, was quantifiable using both plasma-input and reference-tissue modeling, demonstrated graded sensitivity to intravenous naloxone blockade, and showed measurable within-scan displacement after intravenous or intranasal naloxone challenge during bolus-plus-infusion administration. Together, these findings support the feasibility of quantitative and pharmacologically responsive MOR PET with [^18^F]FCFN in NHPs and represent an early translational step from the prior rodent evaluation toward human studies.

Kinetic modeling with metabolite-corrected arterial input functions was used to characterize regional tracer kinetics and quantify total *V*_T_. The two-tissue compartment model fit baseline regional TACs well, with *V*_T_ values following the expected pattern across MOR-enriched and low-binding regions (Figure 3) [40]. The strong agreement between 2TC- and MA1-derived *V*_T_ estimates at baseline supports the robustness of plasma-input quantification (Figure S7A). Under naloxone pretreatment conditions, the lower correspondence likely reflected the compressed regional dynamic range of *V*_T_ after MOR blockade, increasing sensitivity to noise and limiting the identifiability of individual microparameters; post-blockade *k*_3_ and *k*_4_ estimates (Figure S7B and Table S4) should therefore be interpreted cautiously.

Reference-tissue analyses supported prioritization of the cerebellum over visual cortex as the reference region. The cerebellum showed lower uptake, faster washout, and smaller naloxone-associated changes than visual cortex (Figure S8), while cerebellum-based BP_ND_ estimates showed regional correspondence with plasma-input *V*_T_ (Figure 3E and Figure S6E). Together with the minimal changes in cerebellar TAC slope after within-scan naloxone challenge (Figure 5A,B), these findings are consistent with a relatively small specific binding component in the cerebellum, as previously discussed in the [^11^C]CFN literature [17,36,37,40].

The possibility that minor [^18^F]FCFN radiometabolites could contribute to the brain PET signal cannot be excluded from plasma HPLC alone. However, the absence of progressive late retention in regional TACs, together with the observed concordance between plasma-input and reference-tissue measures, argues against a substantial regionally heterogeneous or progressively accumulating radiometabolite contribution to brain quantification. Prior [^11^C]CFN studies similarly identified predominantly polar radiometabolites, including a smaller more lipophilic component, without evidence that cerebral radiometabolite uptake materially compromised quantitative brain PET analysis [5,40]. Comprehensive structural identification of [^18^F]FCFN radiometabolites and direct assessment of their brain penetration were beyond the scope of this initial NHP feasibility evaluation but will be important for subsequent translational development.

The naloxone pretreatment results further demonstrated that the regional [^18^F]FCFN signal was pharmacologically sensitive. Both low- and high-dose intravenous naloxone reduced regional BP_ND_ with greater reductions under the high-dose conditions (Table 1). The high-dose condition produced near-complete reductions in many regions, whereas the low-dose condition preserved the regional rank order. Importantly, marked high-dose blockade was observed in both animals, providing cross-animal support for this principal pharmacologic finding. This graded response suggests that [^18^F]FCFN is sensitive to partial as well as near-saturating MOR blockade, a property relevant to future studies of antagonist engagement.

The bolus-plus-infusion experiments provided proof-of-concept that [^18^F]FCFN can capture dynamic antagonist-induced displacement within a single scan. Bolus-plus-infusion administration produced stable regional uptake over the 60–75 min pre-challenge interval, meeting the operational criterion for an apparent steady-state plateau before naloxone administration at 75 min. Both intravenous and intranasal naloxone challenges produced clear displacement (Figure 5 and Table 2). The nucleus accumbens showed the fastest displacement among the regions examined. Consistent with route-dependent differences in systemic delivery, intravenous naloxone produced more rapid displacement than intranasal naloxone. Nevertheless, displacement after intranasal challenge was clearly observed within the post-challenge acquisition window. Both routes produced more than 50% displacement in high-binding regions by the end of the scan, whereas the intranasal time course was more gradual, consistent with absorption-limited kinetics [41]. These findings demonstrate that [^18^F]FCFN can capture dynamic MOR engagement after both intravenous and intranasal naloxone, with the intranasal experiment providing a particularly relevant proof-of-concept for future studies of route-dependent MOR pharmacodynamics.

One consistent regional observation across scans was slower [^18^F]FCFN washout in the amygdala than in the NAC and other high-binding regions, producing a reproducible NAC–amygdala crossover late in the scan (Figure 2, Figure 3, Figure 4 and Figure 5). This pattern may reflect a combination of regional receptor density, region-specific tissue kinetics, nondisplaceable retention, and partial-volume effects related to the complex anatomy and boundaries of the amygdala. We do not draw a mechanistic conclusion from this observation, but its recurrence across scans suggests that it is unlikely to represent a single-scan artifact and warrants further evaluation in larger datasets, particularly given the role of the amygdala in affective and stress-related dimensions of opioid action.

Several limitations should be considered when interpreting these findings and planning future studies. This study included two rhesus macaques and was designed as an initial NHP radiotracer evaluation rather than a statistically powered between-subject study. The small number of animals limits characterization of inter-animal variability and broader generalizability of the findings. Several pharmacologic conditions were represented by single scans; therefore, observed dose- and route-related differences should be interpreted descriptively and confirmed in larger, adequately powered studies. The cerebellum showed a small naloxone-sensitive component that may bias BP_ND_ estimates downward. Anesthesia may influence physiology, cerebral blood flow, and receptor systems in ways that differ from awake human imaging. Despite these limitations, the observed in vivo properties of [^18^F]FCFN support continued translational evaluation.

If successfully translated to human imaging, [^18^F]FCFN could provide a quantitative tool for addressing clinically relevant gaps in opioid pharmacology that remain difficult to assess directly in vivo. A particularly direct application would be measuring brain MOR engagement after naloxone administration across different doses, routes, and timepoints, complementing the systemic pharmacokinetic and clinical outcome data that currently inform overdose-reversal strategies. Such studies could also test whether interindividual differences in baseline MOR availability influence antagonist engagement and its time course [5,7,9,10]. More broadly, [^18^F]FCFN could support studies of MOR availability and receptor engagement during pharmacotherapy for opioid use disorder, including treatment with buprenorphine or methadone, where residual receptor availability and pharmacologic competition remain incompletely characterized [10]. These applications illustrate how quantitative and dynamically responsive MOR PET with [^18^F]FCFN could extend beyond tracer characterization toward mechanistic studies of opioid pharmacology and treatment in humans.

## 5. Conclusions

[^18^F]FCFN was produced by automated radiosynthesis and demonstrated MOR-consistent regional brain distribution, quantitative outcome measures from plasma-input and reference-tissue modeling, dose-dependent naloxone-sensitive binding, and within-scan displacement after intravenous and intranasal naloxone challenge in rhesus macaques. These findings support further evaluation of [^18^F]FCFN in humans and its potential use in studies of MOR availability, antagonist occupancy, and route-dependent opioid-system pharmacodynamics.

## Figures and Tables

**Table 1:** Regional BP_ND_ values at baseline and after pretreatment with low- or high-dose intravenous naloxone. Percent reductions relative to baseline BP_ND_ are also shown for each blocking dose. BP_ND_ values were estimated using MRTM2 with the cerebellum as the reference region.

Brain Region	Baseline BP_ND_	Low- Blocking BP_ND_	% Change	High- Blocking BP_ND_	% Change
Amygdala	4.59	3.28	−28%	0.18	−96%
Nucleus Accumbens	3.78	2.10	−44%	0.21	−94%
Hypothalamus	2.75	1.87	−32%	0.15	−95%
Thalamus	1.98	1.15	−42%	0.13	−93%
Caudate	1.59	0.77	−52%	0.10	−94%
Anterior Cingulate	1.48	0.64	−57%	0.13	−91%
Putamen	1.30	0.66	−49%	0.18	−86%
Hippocampus	1.07	0.61	−43%	0.11	−90%
Frontal Cortex	0.77	0.34	−56%	0.14	−82%
Pons	0.63	0.39	−38%	0.06	−90%
Visual Cortex	0.30	0.13	−57%	0.13	−58%

**Table 2:** Regional BP_ND_ values before and after within-scan naloxone challenge. For each type of challenge (intravenous vs. intranasal), regional BP_ND_ values are shown from the pre-naloxone (“baseline”; 0–75 min) and post-naloxone (“post-NLX”; 75–140 min) portions of the scan. Percent reductions in regional BP_ND_ are also shown. For each type of challenge, the final column shows the time elapsed between the naloxone challenge (75 min into the scan) and a 50% reduction in regional BP_ND_. In these columns, values >65 indicate that 50% displacement was not reached before scan completion. NAC: Nucleus accumbens.

Brain Region	Intravenous Naloxone				Intranasal Naloxone			
	Baseline BP_ND_	Post-NLX BP_ND_	% Change	50% Displacement Time (min)	Baseline BP_ND_	Post-NLX BP_ND_	% Change	50% Displacement Time (min)
NAC	2.68	0.21	−92%	30	2.30	0.61	−73%	45
Amygdala	2.40	0.21	−91%	40	2.34	0.21	−91%	>65
Hypothalamus	1.93	0.07	−96%	35	1.87	0.24	−87%	60
Thalamus	1.63	0.09	−94%	35	1.37	0.13	−90%	60
Caudate	1.22	0.03	−97%	40	1.00	0.01	−99%	60
Anterior Cingulate	1.17	0.09	−92%	40	0.81	0.02	−97%	>65
Putamen	1.00	0.18	−82%	55	0.75	0.14	−82%	>65
Hippocampus	0.79	0.05	−94%	60	0.54	0.10	−82%	>65
Frontal Cortex	0.69	0.14	−79%	>65	0.49	0.15	−70%	>65
Pons	0.59	0.00	−100%	>65	0.59	0.03	−94%	>65
Motor Cortex	0.35	0.04	−89%	>65	0.22	0.02	−92%	>65
Visual Cortex	0.19	0.11	−41%	>65	0.22	0.14	−36%	>65

## Data Availability

The datasets generated and/or analyzed during the current study are available from the corresponding author upon reasonable request, subject to execution of an appropriate data sharing agreement.

## Supplementary Materials

The following supporting information can be downloaded at https://www.mdpi.com/article/10.3390/pharmaceutics18091141/s1. Figure S1: Automated synthesis cassette configuration for [^18^F]FCFN using the Trasis AllInOne; Figure S2: HPLC purification and analytical characterization of [^18^F]FCFN; Figure S3: HPLC purification and analytical characterization of *p*-^18^F-2; Table S1: Summary and study design; Table S2: Regional standardized uptake value (SUV) metrics from two baseline [18F]FCFN PET scans in the same animal; Figure S4: Volumes of interest (VOI) for regional analysis of [^18^F]FCFN PET data; Figure S5: Baseline *p*-^18^F-2 PET images and time–activity curves; Table S3: Akaike information criteria (AIC) for one- and two-tissue compartment models; Table S4: Two-tissue compartment modeling parameters in baseline and blocked conditions; Figure S6: Two-tissue compartment modeling results following naloxone blocking; Figure S7: Associations between MA1 distribution volumes and 2TC distribution volumes; Figure S8: Reference region evaluation.

## Abbreviations

PET	positron emission tomography
MOR	μ-opioid receptor
FCFN	fluorocarfentanil
V_T_	volume of distribution
BP_ND_	nondisplaceable binding potential
CFN	carfentanil
RCY	radiochemical yield
RCP	radiochemical purity
A_m_	molar activity
BPin	aryl boronic ester precursor
HPLC	high-performance liquid chromatography
*f*_P_	plasma free fraction
MRI	magnetic resonance imaging
TAC	time–activity curve
SUV	standardized uptake value
OSEM	ordered subset expectation maximization
NHP	nonhuman primate
CT	computed tomography
1TC	one-tissue compartment
2TC	two-tissue compartment
AIC	Akaike information criterion
MA1	multilinear analysis 1
MRTM2	multilinear reference tissue model 2
EOB	end of bombardment
EOS	end of synthesis
ROI	region of interest
NAC	nucleus accumbens
ACC	anterior cingulate cortex
IV	intravenous
IN	intranasal

## References

[1] Kieffer B.L., Evans C.J. (2009). Opioid Receptors: From Binding Sites to Visible Molecules in Vivo. Neuropharmacology.

[2] Williams J.T., Ingram S.L., Henderson G., Chavkin C., von Zastrow M., Schulz S., Koch T., Evans C.J., Christie M.J. (2013). Regulation of M-Opioid Receptors: Desensitization, Phosphorylation, Internalization, and Tolerance. Pharmacol. Rev..

[3] Corder G., Castro D.C., Bruchas M.R., Scherrer G. (2018). Endogenous and Exogenous Opioids in Pain. Annu. Rev. Neurosci..

[4] Darcq E., Kieffer B.L. (2018). Opioid Receptors: Drivers to Addiction?. Nat. Rev. Neurosci..

[5] Frost J.J., Douglass K.H., Mayberg H.S., Dannals R.F., Links J.M., Wilson A.A., Ravert H.T., Crozier W.C., Wagner H.N. (1989). Multicompartmental Analysis of [^11^C]-Carfentanil Binding to Opiate Receptors in Humans Measured by Positron Emission Tomography. J. Cereb. Blood Flow. Metab..

[6] Hirvonen J., Aalto S., Hagelberg N., Maksimow A., Ingman K., Oikonen V., Virkkala J., Någren K., Scheinin H. (2009). Measurement of Central M-Opioid Receptor Binding in Vivo with PET and [^11^C]Carfentanil: A Test–Retest Study in Healthy Subjects. Eur. J. Nucl. Med. Mol. Imaging.

[7] Zubieta J.-K., Smith Y.R., Bueller J.A., Xu Y., Kilbourn M.R., Jewett D.M., Meyer C.R., Koeppe R.A., Stohler C.S. (2001). Regional Mu Opioid Receptor Regulation of Sensory and Affective Dimensions of Pain. Science.

[8] Bencherif B., Fuchs P.N., Sheth R., Dannals R.F., Campbell J.N., Frost J.J. (2002). Pain Activation of Human Supraspinal Opioid Pathways as Demonstrated by [^11^C]-Carfentanil and Positron Emission Tomography (PET). Pain.

[9] Zubieta J.-K., Gorelick D.A., Stauffer R., Ravert H.T., Dannals R.F., Frost J.J. (1996). Increased Mu Opioid Receptor Binding Detected by PET in Cocaine–Dependent Men Is Associated with Cocaine Craving. Nat. Med..

[10] Heinz A., Reimold M., Wrase J., Hermann D., Croissant B., Mundle G., Dohmen B.M., Braus D.H., Schumann G., Machulla H.-J. (2005). Correlation of Stable Elevations in Striatal μ-Opioid Receptor Availability in Detoxified Alcoholic Patients With Alcohol Craving: A Positron Emission Tomography Study Using Carbon 11–Labeled Carfentanil. Arch. Gen. Psychiatry.

[11] Johansson J., Hirvonen J., Lovró Z., Ekblad L., Kaasinen V., Rajasilta O., Helin S., Tuisku J., Sirén S., Pennanen M. (2019). Intranasal Naloxone Rapidly Occupies Brain Mu-Opioid Receptors in Human Subjects. Neuropsychopharmacology.

[12] Dubroff J.G., Hsieh C.-J., Wiers C.E., Lee H., Schmitz A., Li E.J., Schubert E.K., Mach R.H., Kranzler H.R. (2025). [^11^C]Carfentanil PET Whole-Body Imaging of μ-Opioid Receptors: A First in-Human Study. J. Nucl. Med..

[13] Kelleher A.C., Pearson T.D., Ramsey J., Zhao W., O’Conor K.A., Bakhoda A., Stodden T., Guo M., Eisenberg S.M., Shah S.V. (2024). Investigation of [^11^C]Carfentanil for Mu Opioid Receptor Quantification in the Rat Brain. Sci. Rep..

[14] Frost J.J., Mayberg H.S., Sadzot B., Dannals R.F., Lever J.R., Ravert H.T., Wilson A.A., Wagner H.N., Links J.M. (1990). Comparison of [^11^C]Diprenorphine and [^11^C]Carfentanil Binding to Opiate Receptors in Humans by Positron Emission Tomography. J. Cereb. Blood Flow. Metab..

[15] Wester H.-J., Willoch F., Tölle T.R., Munz F., Herz M., Øye I., Schadrack J., Schwaiger M., Bartenstein P. (2000). 6-O-(2-[18F]Fluoroethyl)-6-O-Desmethyldiprenorphine ([18F]DPN): Synthesis, Biologic Evaluation, and Comparison with [^11^C]DPN in Humans. J. Nucl. Med..

[16] Reale C., Costanzo G., Cosentino G., Parenti C., Pasquinucci L. (2025). PET Imaging of the Opioid System Using Radioligands: Synthetic Approaches and Translational Applications. Eur. J. Med. Chem..

[17] Lee S.J., Pearson T.D., Dhaynaut M., MacDonagh A.C., Wey H.-Y., Wilks M.Q., Roth B.L., Hooker J.M., Normandin M.D. (2025). Selective Mu-Opioid Receptor Imaging Using 18F-Labeled Carfentanils. J. Med. Chem..

[18] du Sert N.P., Hurst V., Ahluwalia A., Alam S., Avey M.T., Baker M., Browne W.J., Clark A., Cuthill I.C., Dirnagl U. (2020). The ARRIVE Guidelines 2.0: Updated Guidelines for Reporting Animal Research. PLoS Biol..

[19] Feng D., Huang S.-C., Wang X. (1993). Models for Computer Simulation Studies of Input Functions for Tracer Kinetic Modeling with Positron Emission Tomography. Int. J. Bio-Med. Comput..

[20] Wang C., Schroeder F.A., Wey H.-Y., Borra R., Wagner F.F., Reis S., Kim S.W., Holson E.B., Haggarty S.J., Hooker J.M. (2014). In Vivo Imaging of Histone Deacetylases (HDACs) in the Central Nervous System and Major Peripheral Organs. J. Med. Chem..

[21] Schlemmer H.-P.W., Pichler B.J., Schmand M., Burbar Z., Michel C., Ladebeck R., Jattke K., Townsend D., Nahmias C., Jacob P.K. (2008). Simultaneous MR/PET Imaging of the Human Brain: Feasibility Study. Radiology.

[22] Catana C. (2019). Development of Dedicated Brain PET Imaging Devices: Recent Advances and Future Perspectives. J. Nucl. Med..

[23] van der Kouwe A.J.W., Benner T., Salat D.H., Fischl B. (2008). Brain Morphometry with Multiecho MPRAGE. NeuroImage.

[24] Slifstein M., Narendran R., Hwang D.-R., Sudo Y., Talbot P.S., Huang Y., Laruelle M. (2004). Effect of Amphetamine on [18F]Fallypride in Vivo Binding to D2 Receptors in Striatal and Extrastriatal Regions of the Primate Brain: Single Bolus and Bolus plus Constant Infusion Studies. Synapse.

[25] Kao P.-F., Kim S., Wagner H.N., Lever J.R., Ravert H.T., Dannals R.F. (1996). Assessing Neuroreceptor Occupancy by Continuous Infusion of Carbon-11 Labeled Radioligands. Eur. J. Nucl. Med..

[26] Catana C., van der Kouwe A., Benner T., Michel C.J., Hamm M., Fenchel M., Fischl B., Rosen B., Schmand M., Sorensen A.G. (2010). Toward Implementing an MRI-Based PET Attenuation-Correction Method for Neurologic Studies on the MR-PET Brain Prototype. J. Nucl. Med..

[27] Dixon W.T. (1984). Simple Proton Spectroscopic Imaging. Radiology.

[28] Ma J. (2008). Dixon Techniques for Water and Fat Imaging. J. Magn. Reson. Imaging.

[29] Fischl B. (2012). FreeSurfer. NeuroImage.

[30] Cox R.W., Jesmanowicz A. (1999). Real-Time 3D Image Registration for Functional MRI. Magn. Reson. Med..

[31] Ourselin S., Roche A., Subsol G., Pennec X., Ayache N. (2001). Reconstructing a 3D Structure from Serial Histological Sections. Image Vis. Comput..

[32] Modat M., Cash D.M., Daga P., Winston G.P., Duncan J.S., Ourselin S. (2014). Global Image Registration Using a Symmetric Block-Matching Approach. J. Med. Imaging.

[33] McLaren D.G., Kosmatka K.J., Oakes T.R., Kroenke C.D., Kohama S.G., Matochik J.A., Ingram D.K., Johnson S.C. (2009). A Population-Average MRI-Based Atlas Collection of the Rhesus Macaque. NeuroImage.

[34] Rohlfing T., Kroenke C.D., Sullivan E.V., Dubach M.F., Bowden D.M., Grant K., Pfefferbaum A. (2012). The INIA19 Template and NeuroMaps Atlas for Primate Brain Image Parcellation and Spatial Normalization. Front. Neuroinform..

[35] Innis R.B., Cunningham V.J., Delforge J., Fujita M., Gjedde A., Gunn R.N., Holden J., Houle S., Huang S.-C., Ichise M. (2007). Consensus Nomenclature for in Vivo Imaging of Reversibly Binding Radioligands. J. Cereb. Blood Flow Metab..

[36] Frost J.J., Wagner H.N.J., Dannals R.F., Ravert H.T., Links J.M., Wilson A.A., Burns H.D., Wong D.F., McPherson R.W., Rosenbaum A.E. (1985). Imaging Opiate Receptors in the Human Brain by Positron Tomography. J. Comput. Assist. Tomogr..

[37] Hsieh C.-J., Hou C., Lee H., Tomita C., Schmitz A., Plakas K., Dubroff J.G., Mach R.H. (2024). Total-Body Imaging of Mu-Opioid Receptors with [^11^C]Carfentanil in Non-Human Primates. Eur. J. Nucl. Med. Mol. Imaging.

[38] Ichise M., Liow J.-S., Lu J.-Q., Takano A., Model K., Toyama H., Suhara T., Suzuki K., Innis R.B., Carson R.E. (2003). Linearized Reference Tissue Parametric Imaging Methods: Application to [^11^C]DASB Positron Emission Tomography Studies of the Serotonin Transporter in Human Brain. J. Cereb. Blood Flow Metab..

[39] Mansour A., Watson S.J., Herz A., Akil H., Simon E.J. (1993). Anatomical Distribution of Opioid Receptors in Mammalians: An Overview. Opioids.

[40] Endres C.J., Bencherif B., Hilton J., Madar I., James Frost J. (2003). Quantification of Brain μ-Opioid Receptors with [^11^C]Carfentanil: Reference-Tissue Methods. Nucl. Med. Biol..

[41] Grassin-Delyle S., Buenestado A., Naline E., Faisy C., Blouquit-Laye S., Couderc L.-J., Le Guen M., Fischler M., Devillier P. (2012). Intranasal Drug Delivery: An Efficient and Non-Invasive Route for Systemic Administration: Focus on Opioids. Pharmacol. Ther..

