# Supplementary material for: Quantitative μ-Opioid Receptor PET with [18F]FCFN in Nonhuman Primates: Naloxone Blockade and Within-Scan Displacement
Source: Pharmaceutics. 2026 Sep 10;18(9):1141. doi: 10.3390/pharmaceutics18091141 (PMC13610183; doi:10.3390/pharmaceutics18091141)
Supplement: Supplementary file 1 [file pharmaceutics-18-01141-s001.zip › pharmaceutics-4538625-supplementary.pdf]

Supplemental Data

Quantitative μ-opioid receptor PET with [^{18}F]FCFN in nonhuman primates: naloxone blockade and within-scan displacement

Evan Gallagher, Joseph W. Downey, Chi-Hyeon Yoo, Hongping Deng, Jessie Fanglu Fu, Qikai Qin, Irena Bass, Hsiao-Ying Wey, Jacob M. Hooker, Christin Y. Sander†, and So Jeong Lee†,*

[1]Athinoula A. Martinos Center for Biomedical Imaging, Department of Radiology, Massachusetts General Hospital and Harvard Medical School, Charlestown, MA 02129, USA

†C.Y.S and S.J.L share senior authorship.

*Corresponding author: slee178@mgh.harvard.edu (S.J.L)

Section S1: Supplemental Radiochemistry Information

S1.1. Radiosynthesis of [^{18}F]FCFN

[^{18}F]FCFN were produced from the corresponding aryl boronic ester precursor (BPin precursor) using a Trasis AllInOne automated synthesizer (AIO 30) equipped with a modified commercially available synthesis cassette (Fig. 1 and Supplemental Fig 1). [^{18}F]Fluoride (55–85 GBq [1500–2300 mCi]) was produced from enriched [^{18}O]H_2O (2.5 mL, > 98%; Rotem) using an 11 MeV Siemens Eclipse HP cyclotron via the ^{18}O(p,n)^{18}F nuclear reaction. The [^{18}F]fluoride was transferred under argon to the automated synthesis module in a shielded hot-cell. [^{18}F]Fluoride was trapped on a Sep-Pak Accel Plus QMA Light cartridge preconditioned with ethanol (10 mL), aqueous potassium triflate solution (94mg/mL), and water (10 mL). The trapped activity was eluted into the reactor with tetrabuthylammonium triflate (TBAOTf, 15 mg) and tetraethylammonium bicarbonate (TEAB, 2 mg) in ethanol (0.2 mL), water (0.4 mL), and acetonitrile (1 mL). The eluate was azeotropically dried to provide [^{18}F]TBAF. A solution of the BPin precursor (2 mg, 4 µmol, 1 equiv.), tetrakis(pyridine)copper(II) triflate ($Cu(OTf)_2(py)_4$; 12 mg, 18 µmol, 4.5 equiv.), and *n*-BuOH (50 µL) in anhydrous *N*,*N*-dimethylacetamide (DMA, 0.7 mL) was added to the dried [^{18}F]fluoride complex. The reaction mixture was heated at 100 °C for 15 min, quenched with water (2 mL), and diluted with ethanol/water (50:50, v/v; 2 mL) before injection onto the semipreparative HPLC system. Purification was performed on a Phenomenex Luna C18(2) column (250x10 mm, 5 µm) eluted at 5 mL/min with 10 mM ammonium acetate in water/methanol (30:70, v/v). The [^{18}F]FCFN product peak eluted at approximately 18–20 min and was collected for 1–2 min. A portion (2.5 mL) of the collected fraction was diluted with water (17.5 mL) and passed through a Waters Sep-Pak tC18 cartridge preconditioned with ethanol (10 mL) and water (10 mL). This trapping step was repeated three additional times to recover the full purified product fraction. The cartridge was rinsed with water (20 mL), dried under nitrogen for 1 min, and eluted with absolute ethanol (200 proof USP, 1 mL) into an intermediate product vial containing saline (0.9% sodium chloride USP, 29 mL). The formulated product was transferred through a sterile 0.22-µm filter (Millex-GP) into a sterile evacuated vial to yield [^{18}F]FCFN for injection. The final formulated dose was analyzed by analytical HPLC using a Phenomenex Kinetex EVO C18 column (150x4.6 mm, 100 Å, 5 µm) eluted at 2 mL/min with 10 mM ammonium acetate in water/methanol (50:50, v/v). [^{18}F]FCFN eluted at approximately 6–7 min.

S1.2. Radiosynthesis of *p*-^{18}F-2

The nonradioactive reference compound *p*-F-2 and the corresponding aryl boronic ester precursor were synthesized as described [1]. *p*-^{18}F-2 was radiolabeled, purified, and characterized using a procedure analogous to that described for [^{18}F]FCFN in Supplementary Method 1.1. The final dose activity at the end-of-synthesis (EOS) was 1.56 GBq (42.1 mCi), radiochemical yield at EOS (n.d.c.) was 2.2%, molar activity at EOS was 98.05 GBq/µmol (2.65 Ci/µmol), and radiochemical purity was 96.3%.

S1.3. Quality control of radiotracers

Radiochemical purity was determined from integration of the radioactive peaks in the analytical HPLC chromatogram. The pH was measured using pH test stripes (pH 2.0–9.0; VWR). Residual copper was assessed using Quantofix copper test strips (Macherey Nagel). Residual TBA salts were evaluated by spot testing against a tetrabutylammonium bicarbonate standard solution (200 µg/mL), followed by visualization in an iodine chamber. Chemical purity was assessed by analytical UV-HPLC. The nonradioactive carrier mass of radiotracers was quantified from the UV-HPLC peak area using a calibration curve generated with the reference standard, and molar activity was calculated from the measured radioactivity and corresponding molar amount of radiotracers at end of synthesis. Final dose-release criteria were radiochemical purity >90%, PH 6.0–8.0, residual copper <10 mg/L, residual TBA salt <200 µg/mL, and tracer cold mass <1.0 µg/dose.

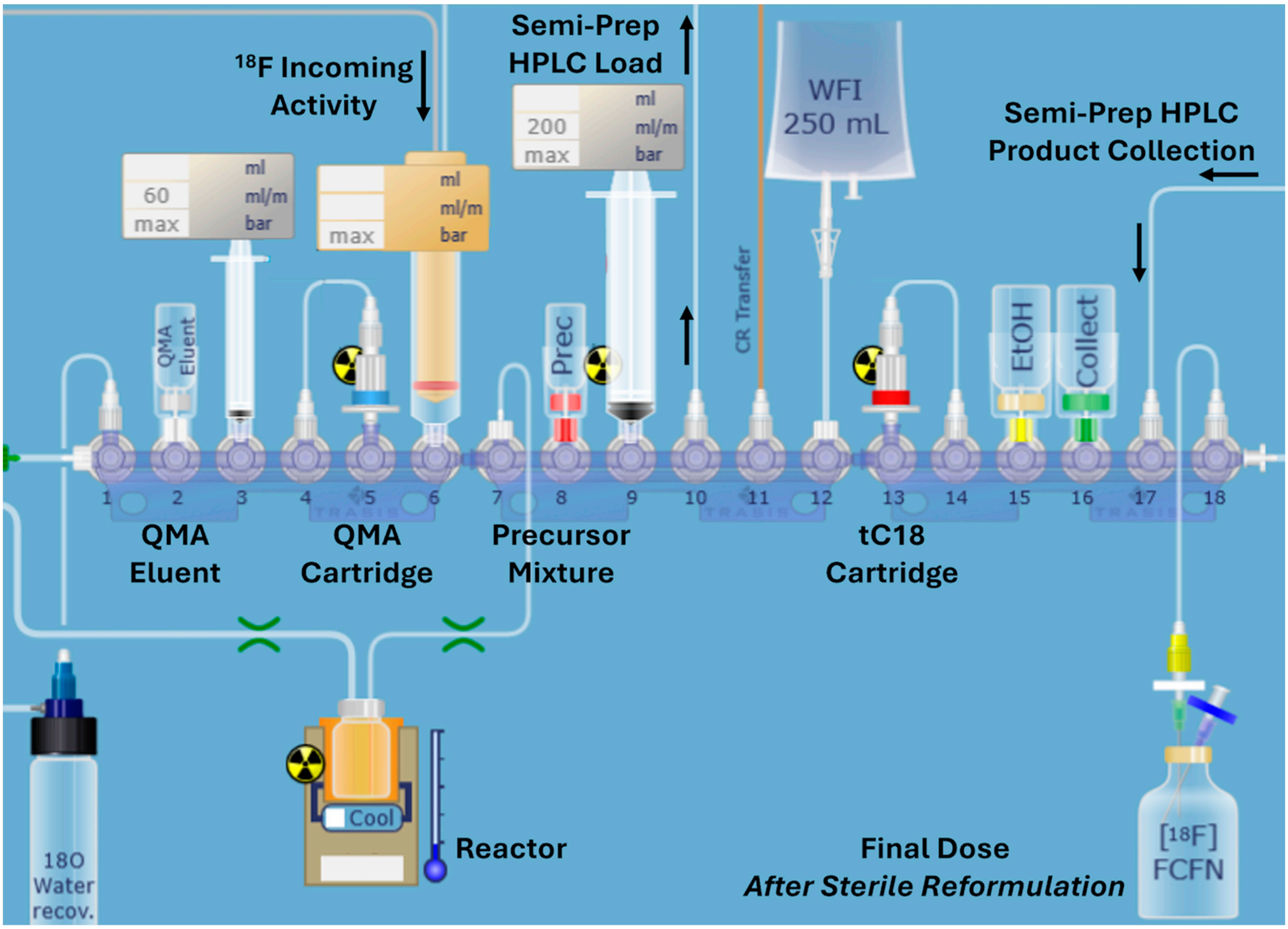

Figure S1. Automated synthesis cassette configuration for [^{18}F]FCFN using the Trasis AllInOne (AIO 30; Ans, Belgium).

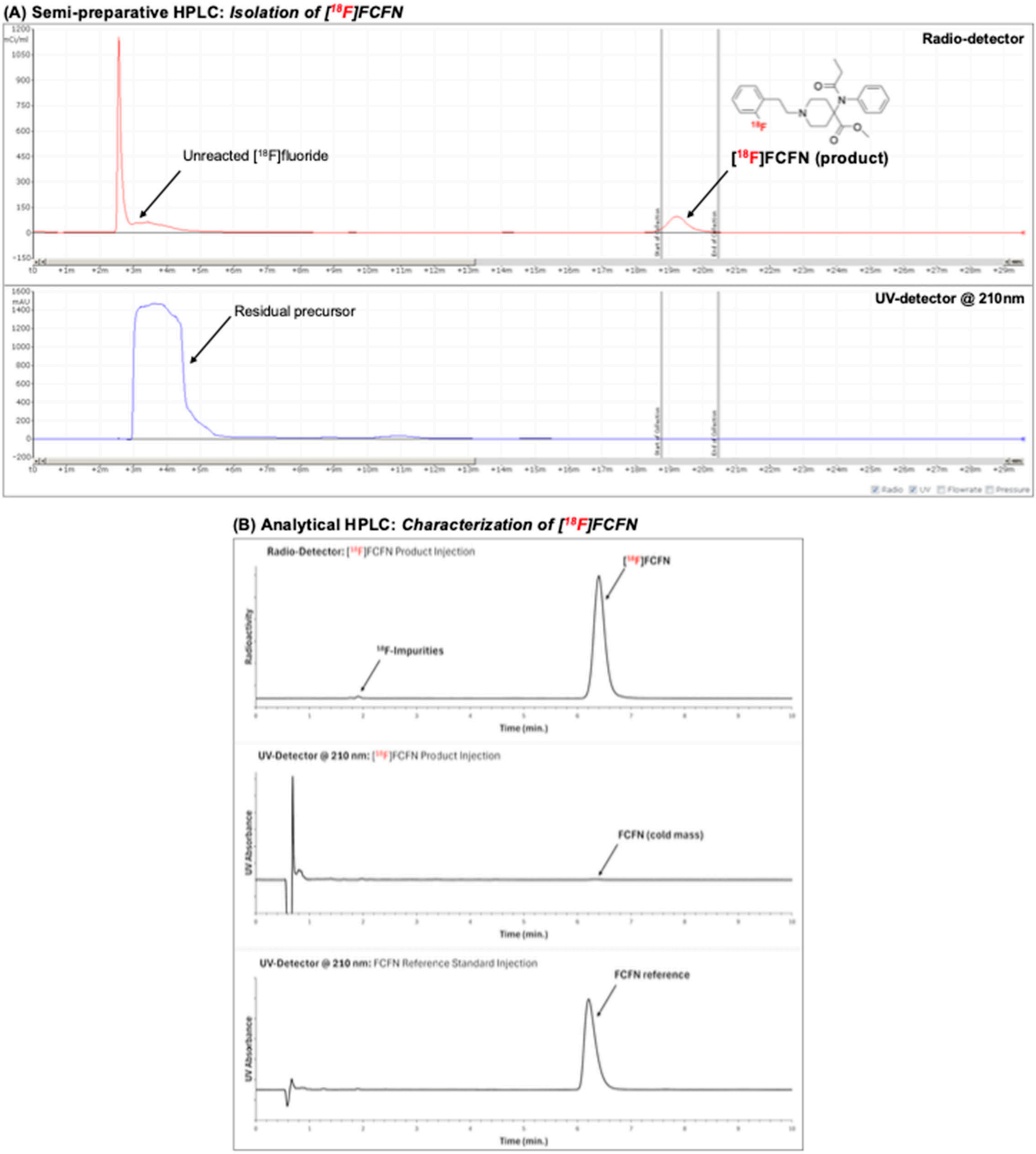

Figure S2. HPLC purification and analytical characterization of [^{18}F]FCFN. (**A**) Semipreparative HPLC chromatogram showing the purified [^{18}F]FCFN product peak at 18.8–20.5 min. (**B**) Analytical HPLC chromatogram demonstrating radiochemical purity (>95%) and the corresponding retention time of nonradioactive FCFN reference standard.

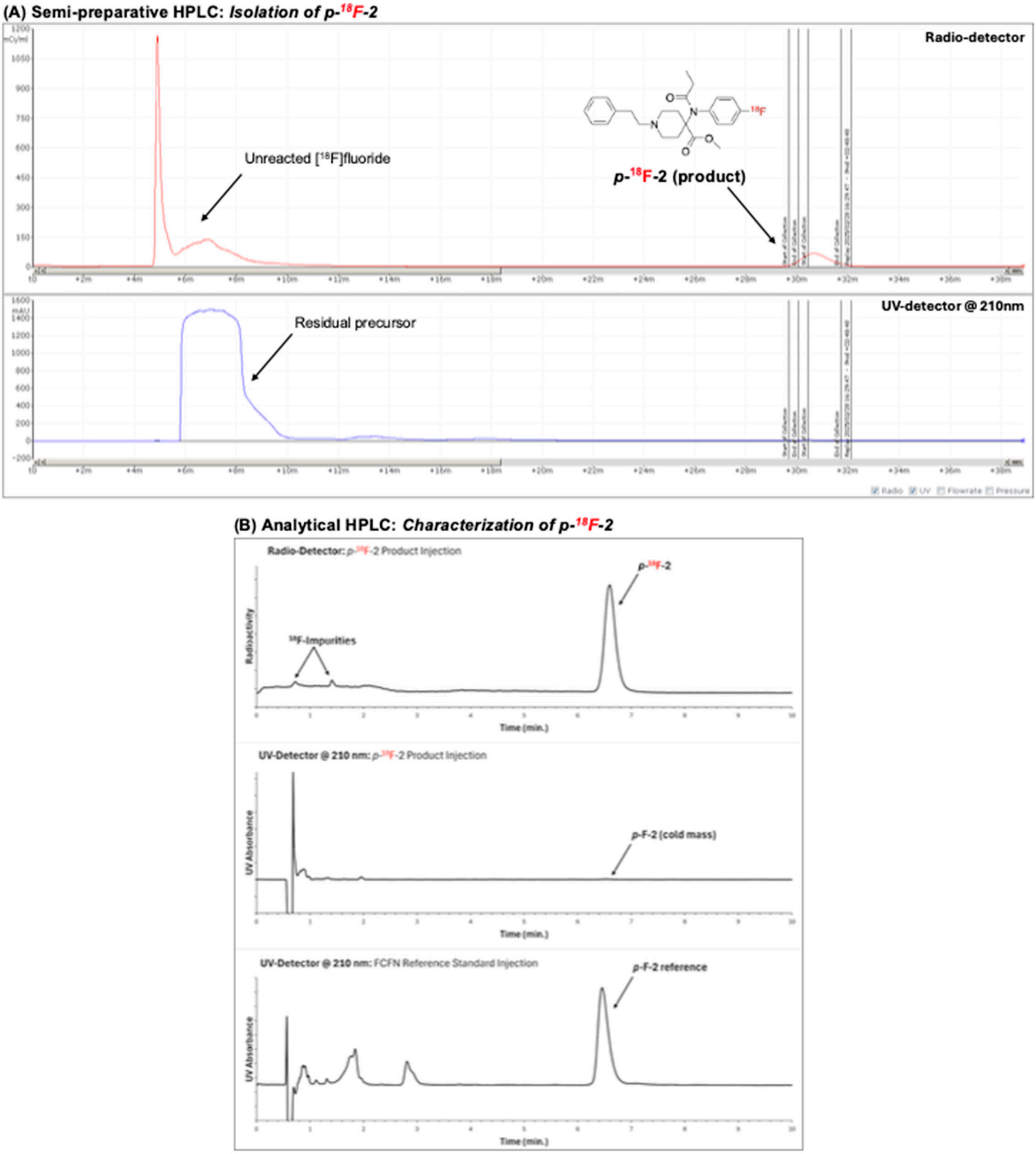

Figure S3. HPLC purification and analytical characterization of *p*-^{18}F-2. (**A**) Semipreparative HPLC chromatogram showing the purified *p*-^{18}F-2 product peak at 30–31.8 min. (**B**) Analytical HPLC chromatogram demonstrating radiochemical purity (>95%) and the corresponding retention time of nonradioactive *p*-F-2 reference standard.

Section S2: Supplemental Information on Animals and Study Design

Two adult male rhesus macaques were used for the PET studies in a series of eight scan sessions. Animal M1 underwent six [^{18}F]FCFN PET scans: two bolus baseline scans, one low-dose intravenous naloxone (0.005 mg/kg) pretreatment scan, one high-dose intravenous naloxone (0.2 mg/kg) pretreatment scan, one bolus-plus-infusion scan with intravenous naloxone (0.2 mg/kg) challenge, and one bolus-plus-infusion scan with intranasal naloxone (0.4 mg/kg) challenge. Animal M2 underwent two PET scans: one supplementary bolus baseline scan with *p*-^{18}F-2 and one high-dose intravenous naloxone (0.2 mg/kg) pretreatment scan with [^{18}F]FCFN. Scan assignment by animal, tracer, administration protocol, and naloxone condition are summarized in Supplemental Table S1.

Scan	Animal ID	Tracer	Condition	Injection Protocol	Injected Dose (KBq)	Injected Mass (μg)	Naloxone Route/Timing	Arterial Sampling
**1**	M1	[^{18}F]FCFN	Baseline	Bolus	69190	0.24	-	Y
**2**	M1	[^{18}F]FCFN	Baseline	Bolus	74000	0.32	-	N
**3**	M1	[^{18}F]FCFN	Low-dose blockade	Bolus	75480	0.22	IV, 10 min *before* tracer injection	N
**4**	M1	[^{18}F]FCFN	High-dose blockade	Bolus	76220	0.22	IV, 10 min *before* tracer injection	N
**5**	M2	[^{18}F]FCFN	High-dose blockade	Bolus	76220	0.09	IV, 10 min *before* tracer injection	Y
**6**	M1	[^{18}F]FCFN	Within-scan displacement	B/I	Bolus: 52540<br>Infusion: 51800	0.24	IV, 75 min *after* tracer injection	N
**7**	M1	[^{18}F]FCFN	Within-scan displacement	B/I	Bolus: 38110<br>Infusion: 37370	0.30	IN, 75 min *after* tracer injection	N
**8**	M2	*p*-^{18}F-2	Baseline	Bolus	88430	0.42	-	N

Table S1. PET scan summary and study design. B/I = bolus-plus-infusion; IV = intravenous; IN = intranasal.

Section S3: Supplemental Information on Data Acquisition and Analysis

S3.1. Naloxone preparation

Pharmaceutical-grade naloxone hydrochloride solution in saline (1 mg/mL; generic equivalent to Narcan; Evzio; McKesson) was used for all pretreatment and within-scan challenge studies. For intravenous administration, the naloxone solution was diluted with sterile saline to a final injection volume of 2 mL for the low-dose (0.005 mg/kg) and high-dose (0.2 mg/kg) conditions. For intranasal administration, naloxone was prepared at the route-specific challenge dose (0.4 mg/kg) in 0.1 mL sterile saline and delivered through intranasal tubing during PET acquisition.

S3.2. Arterial input function and radiometabolite analysis

Twenty-six arterial blood samples were collected during each of two bolus [^{18}F]FCFN PET scans used for arterial-input modeling. For each scan, one 3-mL blood sample was collected before radiotracer injection, and 25 post-injection samples were collected during dynamic PET acquisition. Seventeen 1 mL samples were collected during the first 3 min after injection at 10-sec intervals. Eight additional 3-mL samples were collected at 5, 10, 20, 30, 45, 60, 90, and 120 min after injection. Arterial blood samples were centrifuged at 4000 rpm for 4 min at 4°C to separate plasma. For radiometabolite analysis, 1 mL of plasma was mixed with 1 mL of acetonitrile, vortexed to precipitate plasma proteins, and centrifuged again at 4000 rpm for 4 min. The supernatant was collected, and 1 mL was diluted with 19 mL of deionized water before HPLC analysis. This dilution was used to minimize parent [^{18}F]FCFN breakthrough during the initial enrichment phase. A 5-mL aliquot of the diluted sample was injected onto a column-switching HPLC system consisting of an enrichment column Bond-Elut Online PLRP-S, 15-20 µm, 4.6x12.5 mm; 99% water/1% acetonitrile, 2 mL/min) followed by analytical separation on a C18 column (Agilent Eclipse Plus C18, 3.5 µm, 4.6x150 mm; water/acetonitrile with 0.1% formic acid, 95:5 to 5:95 gradient, 2 mL/min).

The raw HPLC chromatogram data was first decay corrected and baseline corrected before further processing. All radioactive peaks observed in each chromatogram were then individually integrated to give an area under curve (AUC) corresponding to the amount of radioactivity in each peak, with the peak corresponding to the unmetabolized "parent" radiotracer being identified by comparison with a reference injection of a sample of pure [^{18}F]FCFN, and all other peaks being assigned as "metabolites". The parent fraction percentage for each timepoint was then calculated according to the equation: parent fraction (%) = 100*[AUC(parent)/(AUC(parent)+AUC(metabolites))]. Once parent fraction values were generated, a hill function was fitted to the parent fraction data (Fig 3C and S5C) in order to estimate the parent fraction at every timepoint throughout the scan. This fitted Hill function was used to metabolite-correct the plasma activity data from all 25 post-injection blood samples, and a Feng model was used to fit the metabolite-corrected plasma activity data. This model fit was used as the input function for 2TC modeling, which was performed using PMOD version 4.006 (Bruker Corporation, Billerica, Massachusetts, USA)

S3.3. Plasma free fraction measurement

Plasma free fraction (f_p) was measured by ultrafiltration. Before radiotracer injection, a 6-mL blood sample was collected into an EDTA tube and centrifuged for 4 min at 4°C to separate plasma. An aliquot of purified [^{18}F]FCFN in saline was added to 2 mL of plasma, vortexed and incubated for 10 min. at room temperature. Three 50-µL aliquots of the incubated plasma sample were counted to determine total plasma radioactivity concentration. A 1-mL aliquot of the same plasma sample was transferred to a Centrifree ultrafiltration device and centrifuged for 25 min. Three 50 µL aliquots of the ultrafiltrate were collected and counted to determine free radioactivity concentration. f_p was calculated as the ratio of radioactivity concentration in the ultrafiltrate to that in the total plasma sample. The measured f_p of [^{18}F]FCFN was 0.109 ± 0.022 (10.9 ± 2.2%; n=6).

Section S4: Supplemental Figures and Tables

Brain Region	Average SUV 60-120min (g/mL)	Peak SUV (g/mL)	Time to Peak SUV (min)	% of Peak at End of Scan	Time to 50% Washout (min)
Amygdala	3.85	4.18	35.5	89%	>120
Nucleus Accumbens	3.41	4.76	20	63%	>120
Hypothalamus	2.74	3.43	16	69%	>120
Thalamus	2.18	3.45	14	53%	>120
Caudate	1.91	3.55	10	42%	95
Anterior Cingulate	1.67	3.56	10	35%	80
Putamen	1.61	3.67	6.5	35%	70
Hippocampus	1.49	2.89	10	41%	90
Pons	1.15	2.29	8	42%	85
Frontal Cortex	1.15	2.87	6.5	31%	65
Medulla	1.03	1.83	8	47%	105
Motor Cortex	0.86	2.55	6.5	26%	50
Visual Cortex	0.76	3.12	3	19%	30
Cerebellum	0.55	3.32	3	13%	20

Table S2. Regional standardized uptake value (SUV) metrics from two baseline [^{18}F]FCFN PET scans in the same animal. Columns show average regional SUV values from 60-120 min (SUV_{60-120}), peak SUV, time to peak SUV, end-of-scan SUV as percentage of peak, and time to 50% washout from peak.

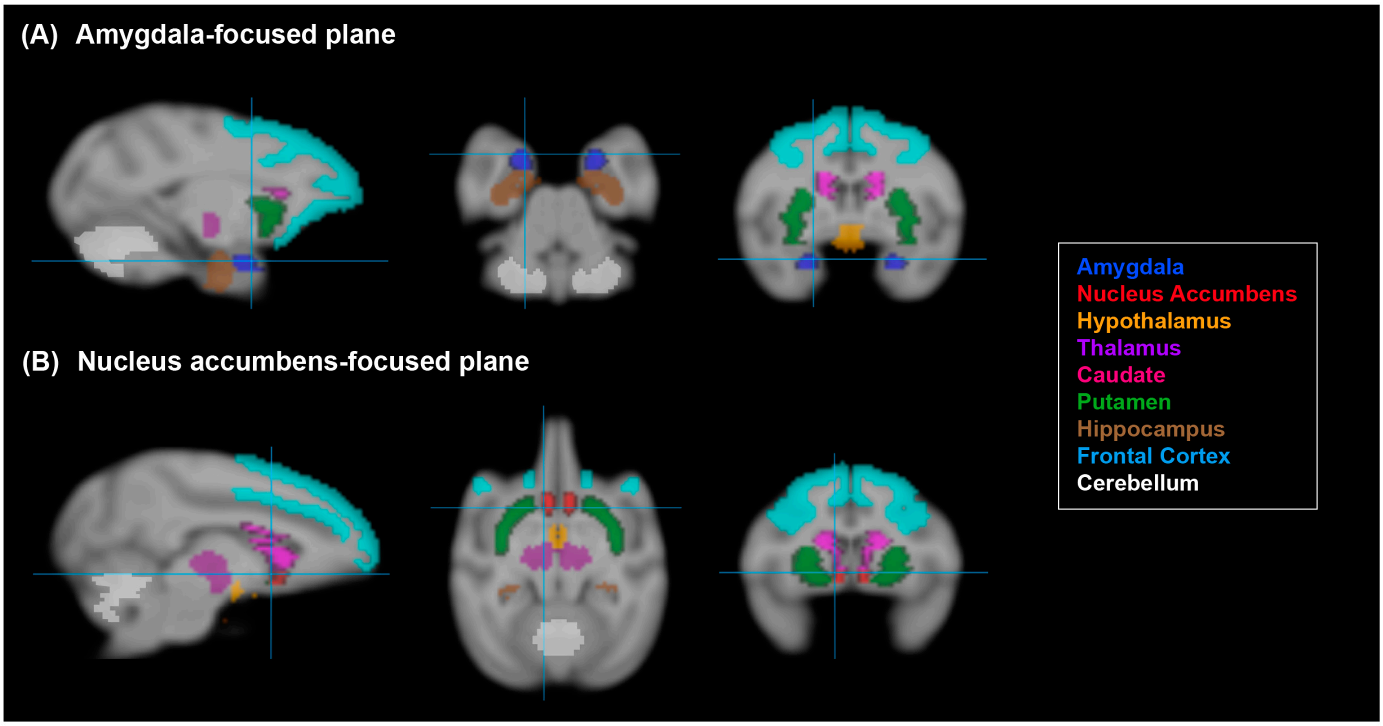

Figure S4. Volumes of interest (VOIs) for regional analysis of [^{18}F]FCFN PET data. (**A**) Nine key VOIs, defined using the NeuroMaps atlas [2], overlaid onto a population-average T1-weighted rhesus macaque MRI template (112RM-SL) [3]. Crosshairs are centered on the left amygdala (identical to the positioning in Figures 2A and S5A), and VOIs are color-coded as shown in the legend. Note that these colors also apply to the corresponding time-activity curves (TACs) in Figures 2C, 3A-B, 4A-C, 5A-B, S5C, and S6A-B. (**B**) Identical VOIs, but with crosshairs centered on the left nucleus accumbens. Note that this view is identical to that shown in Figures 2B and S5B.

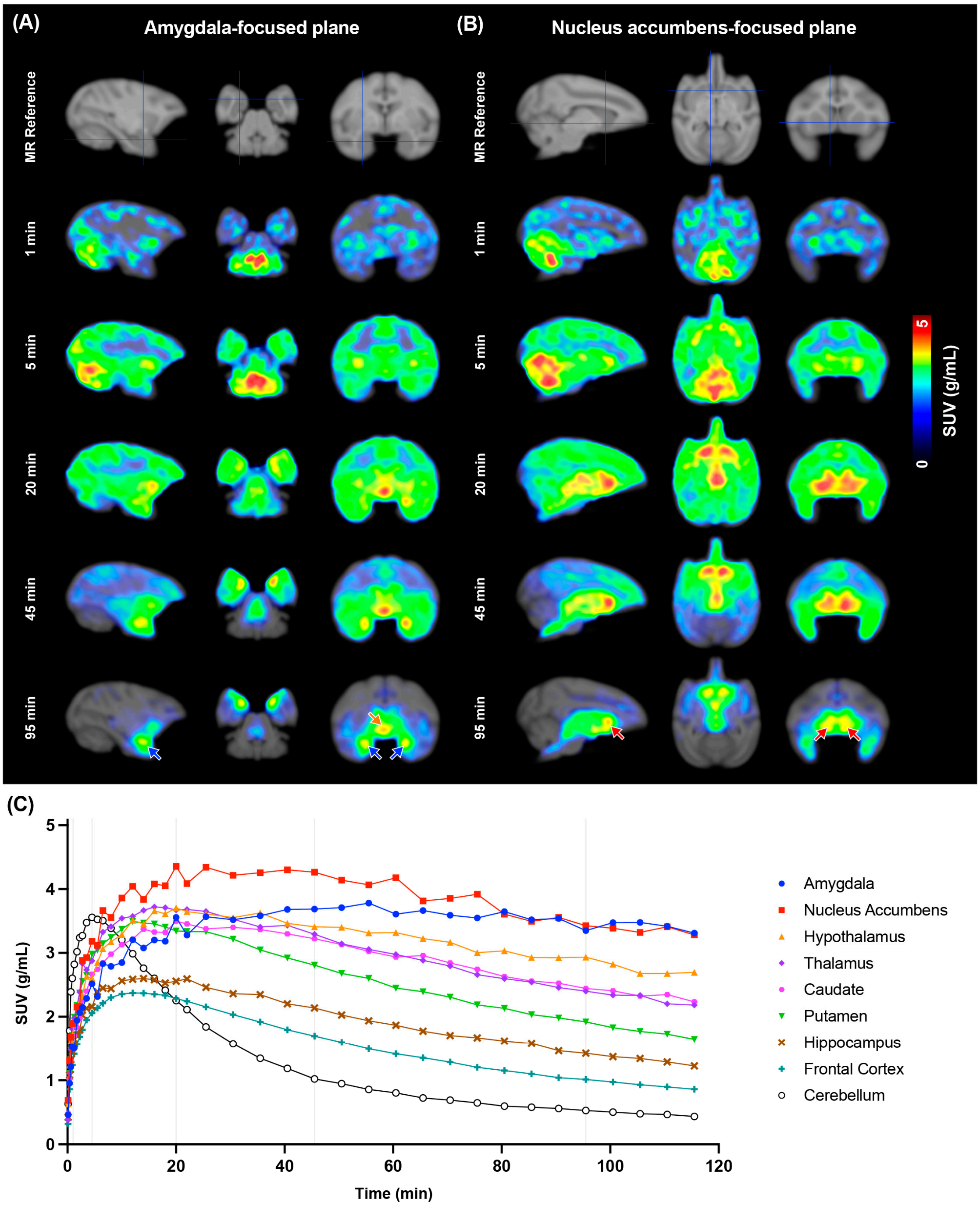

Figure S5. Baseline *p*-^{18}F-2 PET images and time-activity curves. (**A,B**) Average baseline *p*-^{18}F-2 PET images at 1, 5, 20, 45, and 95 minutes post-injection. The anatomical locations and boundaries of the nine key brain regions evaluated in this study are illustrated on a rhesus macaque MRI atlas in Figure S4. In the bottom row of images, blue arrows indicate the amygdala (A), orange arrows indicate the hypothalamus (A), and red arrows indicate the nucleus accumbens (B). In both panels, image colors represent standardized uptake values (SUVs), which are equal to measured radioactivity / (injected dose / body weight). All PET images are masked to the brain and overlaid onto a generic monkey MRI, which is also shown in the top row as an anatomical reference. In these MR images, the blue crosshairs are centered over the left amygdala (A) and left nucleus accumbens (B). (**C**) Time-activity curves (TACs) showing regional SUVs as a function of time. Here, grey vertical lines indicate the timepoints shown in panels A-B.

	Baseline		Blocked	
**Brain Region**	**2 Compartment AIC**	**1 Compartment AIC**	**2 Compartment AIC**	**1 Compartment AIC**
Amygdala	-25.72	41.18	40.75	78.43
Nucleus Accumbens	-14.96	54.44	36.43	61.34
Thalamus	-48.69	38.10	8.67	41.64
Caudate	-41.04	55.70	7.86	58.14
Putamen	-30.10	71.99	28.13	45.08
Hippocampus	-32.97	77.23	43.85	63.50
Frontal Cortex	-63.29	66.71	-16.41	57.57
Motor Cortex	10.43	97.31	33.10	59.52
Anterior Cingulate	-59.90	62.73	4.62	46.84
Hypothalamus	-5.46	60.90	52.24	83.06
Pons	-11.15	78.70	37.64	71.74
Medulla	-20.51	76.77	44.72	79.21
Visual Cortex	24.64	72.17	31.96	71.94
Cerebellum	-0.75	94.68	9.08	33.49

Table S3. Akaike information criteria (AIC) for one- and two-tissue compartment models. AIC values provide a quantitative representation of goodness of model fit, with lower numbers indicating better fits. Two-tissue compartment modeling produced lower AIC values than one-tissue compartment modeling, regardless of brain region or blocking condition. All compartmental modeling was performed in PMOD version 4.006 (Bruker Corporation, Billerica, Massachusetts, USA).

Condition	Region	V_T (mL•cm^{-3})	SE (%)	% Change from Baseline	K_1 (mL•cm^{-3}•min^{-1})	SE (%)	k_2 (min^{-1})	SE (%)	k_3 (min^{-1})	SE (%)	k_4 (min^{-1})	SE (%)
Baseline	Amygdala	29.73	3.01	-	0.26	6.56	0.25	41.68	0.53	27.34	0.019	13.71
	Nucleus Accumbens	18.63	1.55	-	0.41	8.53	0.95	31.08	1.29	16.00	0.031	11.36
	Hypothalamus	15.95	2.19	-	0.31	7.45	0.91	38.25	1.16	27.39	0.025	11.67
	Thalamus	11.92	1.06	-	0.22	7.88	0.17	45.98	0.47	29.44	0.058	15.87
	Caudate	10.49	1.10	-	0.26	1.50	0.13	14.59	0.27	17.84	0.061	6.35
	Anterior Cingulate	9.10	0.71	-	0.29	4.12	0.29	20.54	0.52	14.41	0.063	5.87
	Putamen	8.77	1.17	-	0.26	2.19	0.09	13.49	0.11	20.75	0.055	7.06
	Hippocampus	8.04	1.16	-	0.24	1.93	0.21	12.70	0.29	13.19	0.048	4.80
	Frontal Cortex	6.23	0.66	-	0.22	2.24	0.16	13.47	0.27	14.25	0.075	3.70
	Pons	5.90	1.38	-	0.19	6.59	0.28	28.34	0.39	20.81	0.051	7.59
	Medulla	5.64	1.42	-	0.15	3.12	0.21	18.12	0.29	16.68	0.042	5.83
	Motor Cortex	4.58	1.68	-	0.23	6.33	0.32	25.06	0.36	21.32	0.067	6.88
	Visual Cortex	4.55	3.75	-	0.22	1.97	0.07	7.52	0.01	45.25	0.029	37.06
	Cerebellum	3.39	2.10	-	0.27	1.73	0.13	5.00	0.02	20.20	0.038	15.01
Naloxone Blocking	Amygdala	5.92	43.86	-80%	0.25	2.11	0.09	5.43	0.01	39.68	0.006	118.36
	Nucleus Accumbens	5.39	3.99	-71%	0.31	1.84	0.08	3.93	0.00	17.66	0.010	N/A
	Hypothalamus	5.15	7.81	-68%	0.29	2.83	0.10	8.23	0.01	37.88	0.018	41.12
	Thalamus	5.62	2.84	-53%	0.29	1.38	0.07	2.93	0.00	14.73	0.010	N/A
	Caudate	6.66	49.96	-36%	0.26	1.35	0.08	3.49	0.00	32.09	0.004	126.86
	Anterior Cingulate	5.38	25.15	-41%	0.24	1.36	0.07	3.69	0.00	36.57	0.007	95.05
	Putamen	5.40	3.60	-38%	0.30	1.70	0.08	3.61	0.00	15.92	0.010	N/A
	Hippocampus	4.28	7.81	-47%	0.27	2.38	0.09	6.74	0.01	43.50	0.018	50.60
	Frontal Cortex	4.75	1.96	-24%	0.21	1.01	0.07	2.32	0.01	7.96	0.010	N/A
	Pons	4.83	11.10	-18%	0.23	2.17	0.08	6.57	0.01	37.95	0.014	52.49
	Medulla	3.21	6.47	-43%	0.18	2.44	0.10	7.42	0.01	34.65	0.020	35.72
	Motor Cortex	3.64	3.80	-21%	0.18	1.91	0.07	4.30	0.00	17.53	0.010	N/A
	Visual Cortex	5.02	3.76	10%	0.31	1.83	0.09	3.68	0.01	15.18	0.010	N/A
	Cerebellum	4.24	8.78	25%	0.46	1.17	0.14	2.74	0.00	37.47	0.012	62.76

Table S4. Two-tissue compartment modeling parameters in baseline and blocked conditions. These data correspond to the baseline and blocked results shown in Fig 3. The first two columns show regional distribution volume (V_T) values and corresponding standard errors in each condition, while the third column shows percent changes in V_T in the blocked condition relative to baseline. The following columns show region-specific values of K_1 and $k_{2\text{-}4}$, with applicable standard errors following each rate constant. Note that in the naloxone-blocked condition, some values of k_4 were manually set to 0.01 in order to minimize the applicable Akaike information criterion (AIC). In these cases, standard errors are reported as "N/A" (not applicable). All V_T values were calculated using a 2-tissue compartment model with metabolite-corrected plasma activity as the input function, and all kinetic modeling was performed using PMOD version 4.006 (Bruker Corporation, Billerica, Massachusetts, USA).

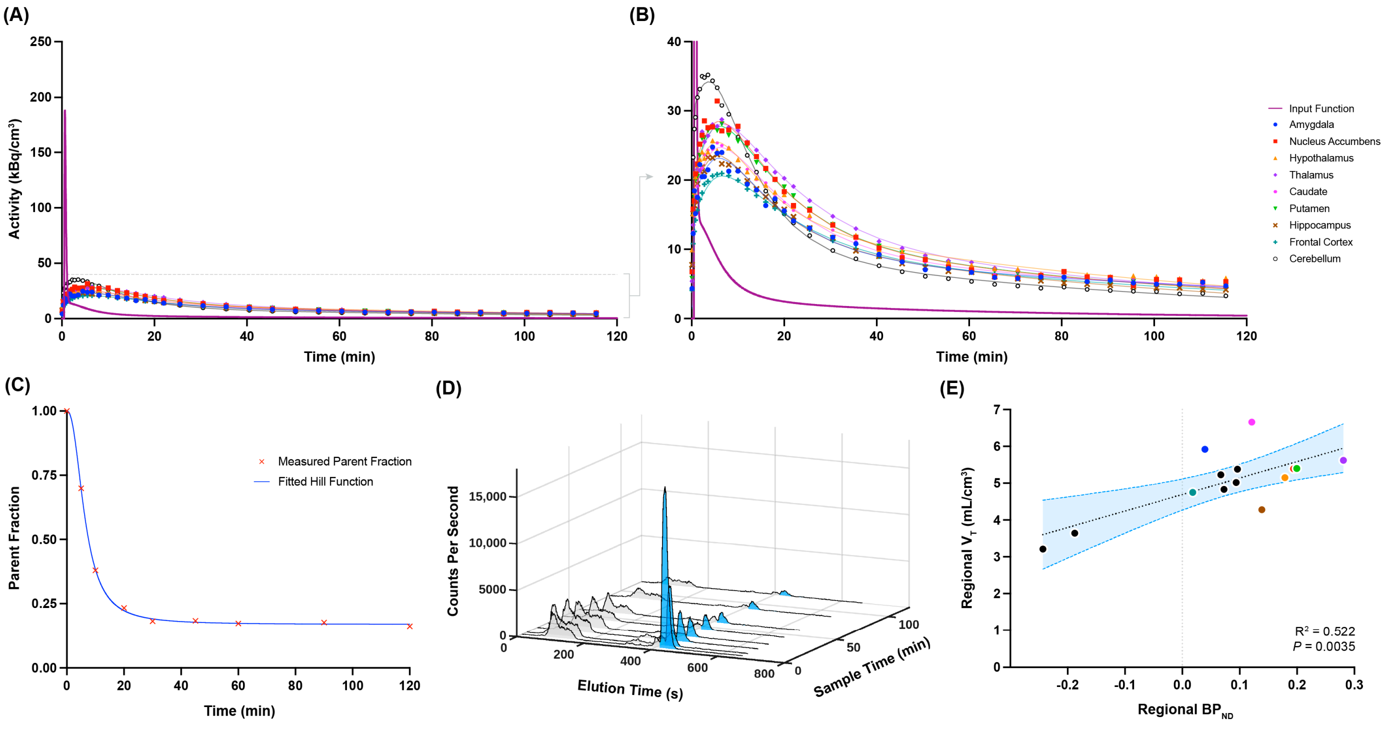

Figure S6. Two-tissue compartment modeling results following naloxone blocking. (**A**) Regional TACs showing the average radioactivity in each ROI as a function of time. Here, the input function (i.e. the concentration of unbound and unmetabolized [^{18}F]FCFN in arterial plasma) is shown as a solid purple line, while regional activity values and model fits are shown as colored icons and curves, respectively. A horizontal gray line at Y=40 indicates the section of this graph that is shown in more detail in panel B. (**B**) Identical TACs as those shown in panel A, but displayed on a graph with a shorter Y-axis. Here, the input function is still shown as a solid purple line, although the peak is not captured. For the non-input function TACs, colored symbols indicate measured activity values, while solid lines indicate two-tissue compartment (2TC) model fits. 2TC modeling was performed in PMOD 4.006, and detailed model parameters are shown in Table S4. (**C**) Parent fraction as a function of time. Nine arterial blood samples were subjected to high performance liquid chromatography (HPLC) to establish parent fraction values at 0, 5, 10, 20, 30, 45, 60, 90, and 120min post-injection (red icons). Subsequently, a Hill function was used to fit the measured parent fraction values and provide a continuous estimate of the [^{18}F]FCFN parent fraction throughout the scan (blue curve). (**D**) HPLC chromatograms for each of the eight post-injection blood samples shown in (C). Here, the parent peak is highlighted in blue. (**E**) Associations between regional distribution volumes (V_T) and nondisplaceable binding potentials (BP_{ND}) following naloxone blocking. V_T values were calculated using 25 metabolite-corrected plasma activity measurements collected throughout each scan, while BP_{ND} values were calculated using the MRTM2 model with cerebellum as the reference region. Note that in this panel, regions are color-coded according to the legend shown in panel B when applicable. Statistical analysis for this figure was performed using GraphPad Prism version 11.0.1 (GraphPad Software, Boston, Massachusetts, USA).

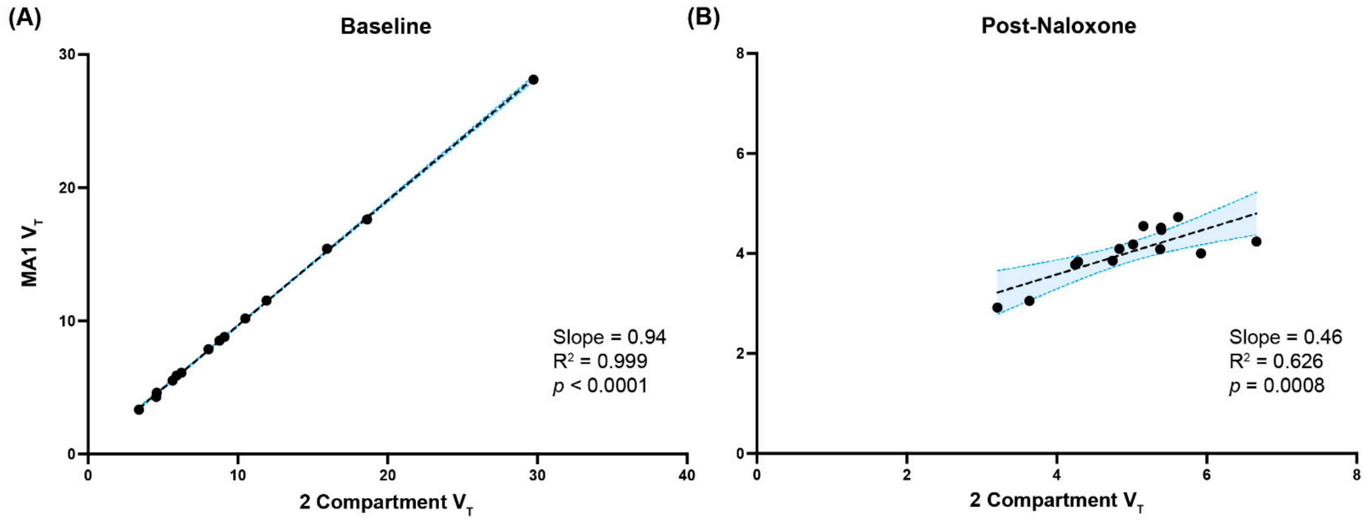

Figure S7. Associations between MA1 distribution volumes and 2TC distribution volumes. (**A**) Average distribution volumes from 14 regions of interest as calculated by the Ichise Multilinear Analysis 1 model ("MA1 V_T", y-axis) and the two-tissue compartment model ("2 Compartment V_T", x-axis) under baseline conditions. Simple linear regression parameters are also shown, with statistical analysis for this figure being performed in GraphPad Prism version 11.0.1 (GraphPad Software, Boston, Massachusetts, USA). (**B**) Equivalent information from the post-naloxone scan. Here, IV naloxone (0.2 mg/kg) was administered 10 min before the scan.

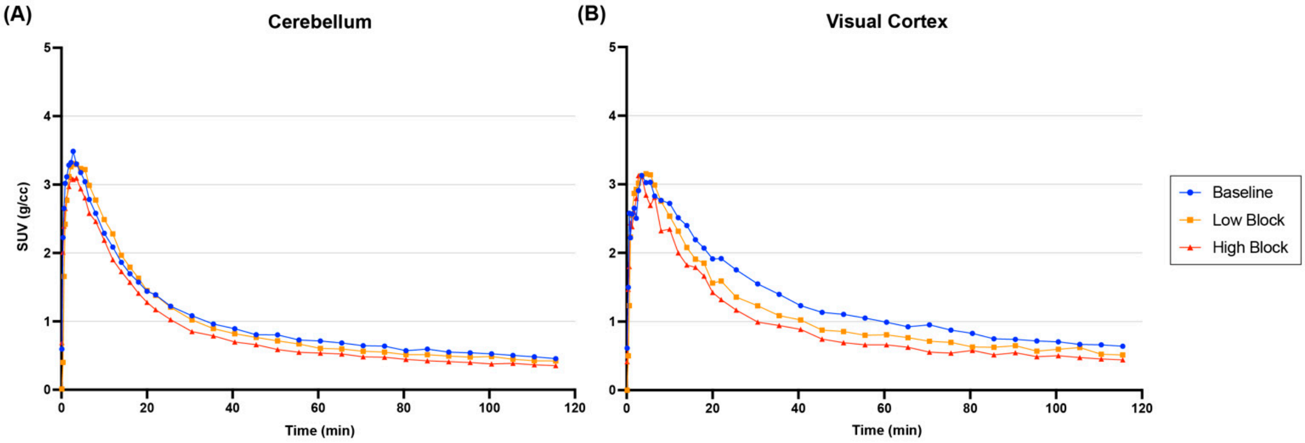

Figure S8. Reference region evaluation. (**A**) Time-activity curves from the cerebellum under baseline (blue), low-block (orange), and high-block (red) conditions. For the low- and high-block conditions, animals were treated with either 0.005 mg/kg (low-block) or 0.2 mg/kg (high-block) of IV naloxone 10 min before the scan. (**B**) Equivalent time-activity curves from the V1 area of visual cortex. In this figure, the same animal was imaged three separate times to generate the baseline/low-block/high-block curves.

References

1. Lee, S.J.; Pearson, T.D.; Dhaynaut, M.; MacDonagh, A.C.; Wey, H.-Y.; Wilks, M.Q.; Roth, B.L.; Hooker, J.M.; Normandin, M.D. Selective Mu-Opioid Receptor Imaging Using 18F-Labeled Carfentanils. *J. Med. Chem.* **2025**, *68*, 1632–1644, doi:10.1021/acs.jmedchem.4c02287.
2. Rohlfing, T.; Kroenke, C.D.; Sullivan, E.V.; Dubach, M.F.; Bowden, D.M.; Grant, K.; Pfefferbaum, A. The INIA19 Template and NeuroMaps Atlas for Primate Brain Image Parcellation and Spatial Normalization. *Front. Neuroinform.* **2012**, *6*, doi:10.3389/fninf.2012.00027.
3. McLaren, D.G.; Kosmatka, K.J.; Oakes, T.R.; Kroenke, C.D.; Kohama, S.G.; Matochik, J.A.; Ingram, D.K.; Johnson, S.C. A Population-Average MRI-Based Atlas Collection of the Rhesus Macaque. *NeuroImage* **2009**, *45*, 52–59, doi:10.1016/j.neuroimage.2008.10.058.
